# Breast cancer diagnosis using support vector machine optimized by improved quantum inspired grey wolf optimization

**DOI:** 10.1038/s41598-024-61322-w

**Published:** 2024-05-10

**Authors:** Anas Bilal, Azhar Imran, Talha Imtiaz Baig, Xiaowen Liu, Emad Abouel Nasr, Haixia Long

**Affiliations:** 1 College of Information Science and Technology, Hainan Normal University, Haikou, 571158 China; 2 Key Laboratory of Data Science and Smart Education, Ministry of Education, Hainan Normal University, Haikou, 571158 China; 3 Department of Creative Technologies, Air University, Islamabad, 44000 Pakistan; 4 School of Life Science and Technology, University of Electronic Science and Technology of China UESTC, Chengdu, Sichuan China; 5 Industrial Engineering Department, College of Engineering, King Saud University, 11421 Riyadh, Saudi Arabia

**Keywords:** Breast cancer, Grey wolf optimization, Support vector machine, Quantum computing, Medical image analysis, Biomedical engineering, Cancer

## Abstract

A prompt diagnosis of breast cancer in its earliest phases is necessary for effective treatment. While Computer-Aided Diagnosis systems play a crucial role in automated mammography image processing, interpretation, grading, and early detection of breast cancer, existing approaches face limitations in achieving optimal accuracy. This study addresses these limitations by hybridizing the improved quantum-inspired binary Grey Wolf Optimizer with the Support Vector Machines Radial Basis Function Kernel. This hybrid approach aims to enhance the accuracy of breast cancer classification by determining the optimal Support Vector Machine parameters. The motivation for this hybridization lies in the need for improved classification performance compared to existing optimizers such as Particle Swarm Optimization and Genetic Algorithm. Evaluate the efficacy of the proposed IQI-BGWO-SVM approach on the MIAS dataset, considering various metric parameters, including accuracy, sensitivity, and specificity. Furthermore, the application of IQI-BGWO-SVM for feature selection will be explored, and the results will be compared. Experimental findings demonstrate that the suggested IQI-BGWO-SVM technique outperforms state-of-the-art classification methods on the MIAS dataset, with a resulting mean accuracy, sensitivity, and specificity of 99.25%, 98.96%, and 100%, respectively, using a tenfold cross-validation datasets partition.

## Introduction

**Figure 1:**
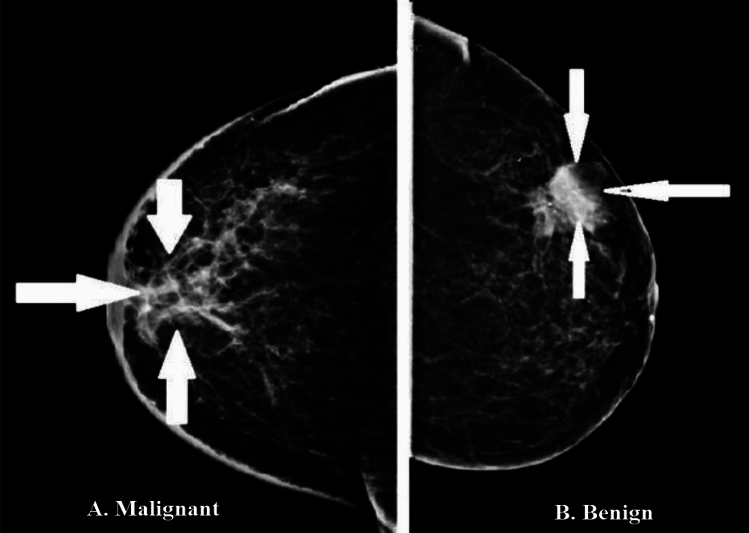
Breast images show benign masses (right) and malignant (left).

Breast Cancer (BC) is a deadly disease that caused 10 million deaths and 19.3 million diagnoses worldwide in 2020^1^. Genetic mutations cause abnormal cell growth, leading to benign or malignant tumors. BC is the 2nd most common cancer globally and the fifth leading cause of death in women. Breast tissues comprise various structures, including connective tissues, blood arteries, lymph nodes, and vessels. Figure 1 illustrates the female breast anatomy. BC can be invasive or non-invasive and is often diagnosed when abnormal breast cells grow uncontrollably, forming tumors. It can spread to other organs through blood vessels, but malignant cells typically remain separate from the cancer. BC has different sub-types based on morphology, form, and structure^2^. In the past decade, mammography-based breast screening has helped diagnose breast lesions and reduce mortality rates by detecting cancer early. Mammography involves imaging the same breast from two angles: the Medio lateral oblique (MLO) and the craniocaudally (CC). Breast density is classified into four categories: fatty, scattered, heterogeneously dense, and highly dense, using the recommended lexicon. Mammography can also classify BC as a mass based on its appearance, aiding in identifying BC^3^.

BC is generally classified as benign or malignant in mammography, as shown in Fig. 1. On mammography, masses that appear as grey to white pixel intensity values are the primary clinical signs of cancer. Within breast regions, breast masses vary in intensity, distribution, shape (lobed, irregular, round, oval), and margins (auricular, obscure, circumscribed), increasing the potential for misdiagnosis. Malignant breast Masses are characterized by irregularly shaped tumors with vague and indistinct boundaries, whereas benign tumors are usually dense, well-defined, demarcated, and roughly spherical. Hidden features near aggregates are essential for BC research. On mammography, benign calcifications are classified as large rods, vascular rough, or popcorn. In contrast, malignant calcifications of the breast are classified as diffuse focal, linear, clustered, amorphous, and segmented^4^.

Breast lesion detection, localization, and grading are often performed manually in mammography, which is time-consuming and dependent on the radiologist's competence and fatigue level. The large number of mammography images produced daily increases the burden on radiologists and the misdiagnosis rate. As a result, the development of computer-aided diagnosis (CAD) systems can significantly reduce the workload of radiologists and improve diagnostic accuracy. CAD helps radiologists distinguish between normal and abnormal tissue and diagnose pathological conditions. Automated diagnostic systems for mammography images must extract regions of interest (ROI) and classify them as normal, benign, and malignant tissue. This is very difficult because the calcifications and masses vary in shape and texture, and the occurrence of blood vessels and muscle fibers compromises accurate detection. These factors make finding competent patterns very difficult.

Identifying a research gap, this study observes that the current diagnostic methods for BC, although improved by CAD systems, still fall short in accuracy and reliability, especially in distinguishing between benign and malignant tumors across varied breast tissue types. This limitation underscores the necessity for a novel computational model to enhance breast cancer classification with greater precision. The improved quantum-inspired binary Gray Wolf Optimizer (IQI-BGWO) combined with a Support Vector Machine (SVM) is proposed to bridge this gap, aiming to advance the classification accuracy beyond the capabilities of current methodologies.

To address this problem, this paper aims to develop a novel, improved quantum-inspired binary Gray Wolf algorithm (IQI-BGWO) and a support vector machine (SVM) to generate an accurate computational BC classification strategy. The binary grey wolf optimization (BGWO) algorithm improves classification accuracy. Many methods have been used to diagnose BC. These include Neural Networks (NN)^5^, Artificial Metaplasticity Neural Networks (AMMLP)^6^, Decision Tree (DT)^7^, deep belief networks^8^, hidden Markov model^9^, K-Nearest Neighbors (KNN), and SVM^10,11^ are included. Hybridization of optimization algorithms and SVMs and Artificial Neural Networks (ANNs) has emerged as a valuable tool for solving today's complex problems such as medical image classification^12–14^ and tumor diagnosis^15,16^. The effectiveness of a classification technique depends on the parameters used. Text, trees, and images are examples of high-dimensional, semi-structured, or unstructured data for which SVMs work particularly well. Its kernel technology is a potent property that allows complex problems to be solved using appropriate kernel functions. SVMs do not find local optima comparable neural networks, but they have less risk of overfitting and outperform ANN models. Although the SVM classification algorithm has proven advantageous, it has limitations in practical applications when choosing the optimal kernel value. This study uses an optimization strategy to determine the optimal SVM parameters to solve this problem. The best parameters for classification accuracy are chosen. SVM performance is affected by variables such as RBF kernel " σ " and error penalty "C." In this study, BGWO and IQI-BGWO are combined with her SVM to develop an automated BC classification approach that increases the accuracy of BC detection by choosing the optimal SVM parameters.

Recent studies have shown that the natural evolution and behavior of various organisms, including animals, insects, birds, and marine life, influence nature-inspired algorithms. These organisms face various search and optimization challenges, including foraging and finding food, and often relying on herd activity to accomplish specific tasks. Computer scientists can use nature-inspired algorithms to resolve complex optimization issues like the selection of features. This paper focuses on the Gray Wolf Optimizer (GWO)^17^, a new algorithm for optimization that simulates grey wolves' hunting and leadership techniques. Two forms of GWO algorithm are proposed binary and stochastic variants^18^.

Moreover, there have been substantial advancements in feature selection and biomarker identification in computational biology. One study introduces a unique algorithm that melds evolutionary computing with machine learning to identify biomarkers accurately. Another research effort showcases a method that combines mutual information with the Binary Grey Wolf algorithm, aiming to boost the efficiency of feature selection. An advanced ensemble model that integrates Grey Wolf Optimization with deep learning has also been developed, specifically designed to enhance the analysis of microarray cancer datasets. These studies collectively represent significant progress in refining computational techniques for biological data analysis^19–21^. This paper proposes an improved combination between binary and quantum-inspired GWOs to address the selection of feature problems and explore the potential of combining quantum computation with nature-inspired algorithms.

Quantum computing (QC) and nature-inspired algorithms have demonstrated the capacity to tackle complex problems with straightforward operations and procedures. QC typically utilizes parallel quantum processing and probabilistic ways to represent quantum data, including the Grover searching algorithm, which decreases the search time within an organized database with N items to $O\sqrt{N}$^22^. The brief factorization algorithm constitutes another quantum algorithm that utilizes quantum operations to tackle factorization problems more quickly. Some quantum operations, such as qubit representation and rotation operations, can also be approximated or implemented on classical hardware, leading to the rise of quantum (Q) inspired algorithms combining Q concepts with classical algorithms to enhance problem-solving performance. Quantum-inspired algorithms are present in numerous disciplines of computation. The positive aspects of randomness and the heuristic advantage of nature-inspired algorithms can be coupled with parallelism on the quantum side by combining them with quantum operations. However, the impact of quantum operations on selecting features utilizing nature-inspired algorithms is inadequately understood. While numerous quantum-inspired algorithms have been devised to address various engineering and computing problems^23–26^. Some have been used to select features^27,28^. Contributions of this study include:

- Identified the challenges in breast cancer diagnosis, emphasizing the limitations of existing manual methods and the need for automated solutions.
- Developed a novel computational strategy for breast cancer classification by hybridizing the improved quantum-inspired binary Gray Wolf Optimizer (IQI-BGWO) with a Support Vector Machine (SVM).
- Addressed the limitations of traditional optimization algorithms such as Particle Swarm Optimization (PSO) and Genetic Algorithm (GA) in the context of breast cancer classification.
- Demonstrated the efficacy of the proposed IQI-BGWO-SVM approach on the MIAS dataset, showcasing superior performance over state-of-the-art classification methods.
- Investigated the use of IQI-BGWO-SVM for feature selection, providing insights into its potential applications beyond classification.

The organization of this paper is as follows: Section "Background" provides an introduction to quantum computing and nature-inspired algorithms, establishing the background for the subsequent discussion. Section "Related work" delves into the specific case study and outlines its findings. The proposed methodology and the corresponding experimental results are detailed in Sections "Material and methods" and "Experimental results", respectively. Section "Conclusion" is dedicated to the discussion of the obtained results. Finally, the last section, serves as a conclusion, summarizing and concluding the key insights presented throughout the paper.=

## Background

### Quantum computing

In QC, a qubit is the basic unit of storage and information, analogous to a classical bit in classical computing. However, unlike classical bits that can only hold a 0 or 1 state, qubits can have a superposition of both states, with specific probabilities assigned to each state. Mathematically, a qubit |$\varphi$〉 represents a linear combination of the |0〉 and |1〉 states, where |0〉 represents the ground state and |1〉 represents the excited state. The probabilities of measuring each state are given by the square of their corresponding coefficients in the linear combination, and the coefficients must satisfy the normalization condition, which requires the sum of their squares to equal 1. Qubits are the building blocks of quantum algorithms and allow for exponentially faster computation for specific tasks, such as factoring large numbers or searching unsorted databases^29^

1 $$ \left| \varphi  \right\rangle  = x\left| 0 \right\rangle  + y\left| 1 \right\rangle  $$

The coefficients a along with b are complex numbers that must satisfy the equation |$x$|2 +|$y$|2 = 1, where |$x$|2 represents the likelihood of locating the quantum-bit $|\varphi \rangle$ in the state |0〉, and |$y$|2 represents the likelihood of locating the quantum-bit $|\varphi \rangle$ in the state |1〉.

In quantum computing, operators or gates execute logical and mathematical computations on the qubits represented by vectors, using operators or gates. These operators can be comprehended through their matrices, illustrating how a quantum system can shift from one state to another. There are four fundamental quantum operators known as Pauli operators: P (the identity operator), X (also termed the NOT operator), Y (the scalar product operator), and Z (the quaternion product operator). Pauli operators and their associated matrices are summarized in Table 1. Other quantum gates, including the Toffoli gate, Feynmann gate, Fredkin gate, Swap gate, and Peres gate, are more intricate. Another form of quantum gate, rotation gates, entails rotating a qubit around the X, Y, or Z axis. These rotations produce, respectively, the x-gate, y-gate, and z-gate. These rotation gates' matrices can also be represented mathematically^30^.

**Table 1:** Q-Gates along with respective matrices.

Gate name	Matrix
I-gate	$$\left(\begin{array}{cc}1& 0\\ 0& 1\end{array}\right)$$
X-gate	$$\left(\begin{array}{cc}0& 1\\ 1& 0\end{array}\right)$$
Y-gate	$$\left(\begin{array}{cc}0& -i\\ i& 0\end{array}\right)$$
Z-gate	$$\left(\begin{array}{cc}1& 0\\ 0& -1\end{array}\right)$$

Integrating classical algorithms with quantum rotation matrices has led to the development of quantum-inspired algorithms. This study employed rotation gates, as shown in Eq. (3), which regulate the GWO update stages. A quantum-inspired algorithm is proposed to study the effect of quantum operations along with nature-inspired algorithms on feature selection.

2 $$x\_gate\left( \theta \right) = \left( {\begin{array}{*{20}c} {cos\left( \theta \right)} & { - isin\left( \theta \right)} \\ {sin\left( \theta \right)} & {cos\left( \theta \right)} \\ \end{array} } \right)$$

3 $$y_{gate\left( \theta \right)} = \left( {\begin{array}{*{20}c} {cos\left( \theta \right)} & { - sin\left( \theta \right)} \\ {sin\left( \theta \right)} & {cos\left( \theta \right)} \\ \end{array} } \right)$$

4 $$z\_gate\left( \theta \right) = \left( {\begin{array}{*{20}c} {e^{{ - \left( {i\theta } \right)}} } & 0 \\ 0 & {e^{{\left( {i\theta } \right)}} } \\ \end{array} } \right)$$

### Nature-inspired algorithms

In recent years, there has been a proliferation of nature-inspired proposals and implementations in various technological contexts. Derviş Karaboa's Artificial Bee Colony (ABC) Algorithm was motivated by the swarming behavior of honey bees and was developed to address problems in numerical optimization.

Seyed Mirjalili proposes that the Grey Wolf Optimizer (GWO)^17^ is based on encircling the prey. Similarly, the Elephant Search Algorithm (ESA) is based on how elephants search for things. Male and female elephants are separated into groups, each searching specific areas. The evolution of microalgae served as the inspiration for the development of the Artificial Algae Algorithm (AAA), which was published in^31^. The Fish Swarm Algorithm (FSA)^32^ is inspired by the method used by fish colonies to find food. The cases mentioned above and others constitute a new category of nature-inspired algorithms.

To elaborate on the choice of the Grey Wolf Optimizer (GWO), interest was drawn to its unique strategy of mimicking the pack behavior of grey wolves, effectively balancing exploration and exploitation in the search space. GWO's proven efficacy in diverse optimization contexts made it a compelling choice. Additionally, empirical tests were performed to validate the selection of GWO. A comprehensive comparative analysis was conducted, featuring the Quantum Grey Binary Grey Wolf Optimizer (Q-GBGWO) against well-established bio-inspired optimization algorithms like GWO, Particle Swarm Optimization (PSO), and Genetic Algorithm (GA). Through benchmark functions, the effectiveness of Q-GBGWO was assessed and compared, providing empirical evidence of its optimization capabilities relative to traditional bio-inspired techniques and enriching the understanding of its potential to address complex optimization challenges effectively.

GWO is a meta-heuristic optimization algorithm first introduced by Mirjalili et al.^17^ in 2014. The social hierarchy and hunting behavior of grey wolves in the wild inspires it. In each of the GWO algorithm's iterations, the 3 best candidate solutions are designated as α, β, and δ. These three wolves act as leaders and guide the rest of the population toward the most prosperous regions of the search space. The remaining wolves are called omega and are tasked with supporting α, β, and δ in hunting and attacking prey. Working together in a hierarchical social structure, the wolves can efficiently explore the search space and find optimal solutions.

The empirical evaluation of GWO through comparative analysis underscores its suitability for specific optimization problems. However, it is crucial to acknowledge the No Free Lunch (NFL) theorem in this context, which states that no single optimization algorithm can solve all problems optimally. This theorem necessitates exploring and experimenting with various optimizers to ascertain their effectiveness in different scenarios.

GW encirclement behavior can be represented analytically with the help of the following eqs:$\mathop{X}\limits^{\rightharpoonup}$

5 $$\begin{array}{*{20}c} {\mathop{K}\limits^{\rightharpoonup} = \left| {\mathop{C}\limits^{\rightharpoonup} {*}\mathop{X}\limits^{\rightharpoonup} _{p} .\left( t \right) - \mathop{X}\limits^{\rightharpoonup} \left( t \right)} \right|} \\ {\mathop{X}\limits^{\rightharpoonup} \left( {t + 1} \right) = \mathop{X}\limits^{\rightharpoonup} _{p} .\left( t \right) - \mathop{A}\limits^{\rightharpoonup} *\vec{K}} \\ \end{array}$$

whereas $t$ specifies the present iteration, $\mathop{A}\limits^{\rightharpoonup} = 2\mathop{a}\limits^{\rightharpoonup} .\mathop{r}\limits^{\rightharpoonup} _{1} - \mathop{a}\limits^{\rightharpoonup}$,$\mathop{C}\limits^{\rightharpoonup} = 2\mathop{r}\limits^{\rightharpoonup} _{2}$, $\mathop{X}\limits^{\rightharpoonup} _{p}$ Is the prey's positional vector, $\mathop{X}\limits^{\rightharpoonup}$ Is the GW's positional vector, $\mathop{a}\limits^{\rightharpoonup}$ is progressively reduced, coming from 2 to 0, and $\mathop{r}\limits^{\rightharpoonup} _{1}$, $\mathop{r}\limits^{\rightharpoonup} _{2}$ are random vectors in the interval [0,1] . The subsequent equations are proposed to model the foraging behavior of grey wolves.

6 $$\begin{gathered}   \overset{\lower0.5em\hbox{$\smash{\scriptscriptstyle\rightharpoonup}$}} {K} _{\alpha }  = \left| {\overset{\lower0.5em\hbox{$\smash{\scriptscriptstyle\rightharpoonup}$}} {C} _{1} *\overset{\lower0.5em\hbox{$\smash{\scriptscriptstyle\rightharpoonup}$}} {X} _{\alpha }  - \overset{\lower0.5em\hbox{$\smash{\scriptscriptstyle\rightharpoonup}$}} {X} } \right| \hfill \\   \overset{\lower0.5em\hbox{$\smash{\scriptscriptstyle\rightharpoonup}$}} {K} _{\beta }  = \left| {\overset{\lower0.5em\hbox{$\smash{\scriptscriptstyle\rightharpoonup}$}} {C} _{2} *\overset{\lower0.5em\hbox{$\smash{\scriptscriptstyle\rightharpoonup}$}} {X} _{\beta }  - \overset{\lower0.5em\hbox{$\smash{\scriptscriptstyle\rightharpoonup}$}} {X} } \right| \hfill \\   \overset{\lower0.5em\hbox{$\smash{\scriptscriptstyle\rightharpoonup}$}} {K} _{\delta }  = \left| {\overset{\lower0.5em\hbox{$\smash{\scriptscriptstyle\rightharpoonup}$}} {C} _{3} *\overset{\lower0.5em\hbox{$\smash{\scriptscriptstyle\rightharpoonup}$}} {X} _{\delta }  - \overset{\lower0.5em\hbox{$\smash{\scriptscriptstyle\rightharpoonup}$}} {X} } \right| \hfill \\  \end{gathered}$$

7 $$\begin{array}{*{20}c} {\mathop{X}\limits^{\rightharpoonup} _{1} = \mathop{X}\limits^{\rightharpoonup} _{\alpha } - \mathop{A}\limits^{\rightharpoonup} _{1} *\vec{K}_{\alpha } } \\ {\mathop{X}\limits^{\rightharpoonup} _{2} = \mathop{X}\limits^{\rightharpoonup} _{\beta } - \mathop{A}\limits^{\rightharpoonup} _{2} *\vec{K}_{\beta } } \\ {\mathop{X}\limits^{\rightharpoonup} _{3} = \mathop{X}\limits^{\rightharpoonup} _{\delta } - \mathop{A}\limits^{\rightharpoonup} _{3} *\vec{K}_{\delta } } \\ \end{array}$$

8 $$\mathop{X}\limits^{\rightharpoonup} \left( {t + 1} \right) = \frac{{\mathop{X}\limits^{\rightharpoonup} _{1} + \mathop{X}\limits^{\rightharpoonup} _{2} + \mathop{X}\limits^{\rightharpoonup} _{3} }}{3}$$

**Figure 2:**
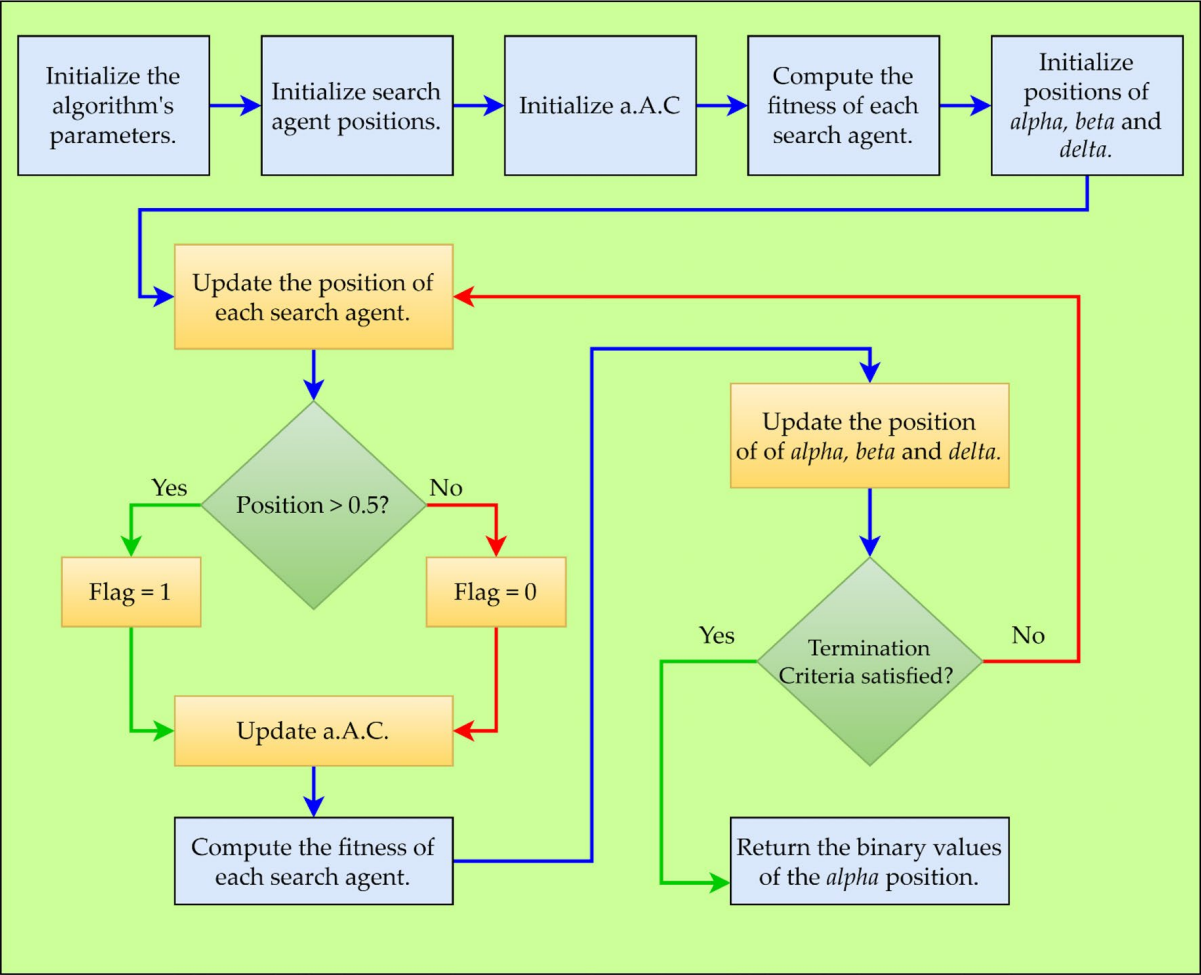
Flowchart of BGWO.

Figure 2 depicts the BGWO as a flowchart. Every GW in the BGWO algorithm has a flag vector that is identical in length to the data set. According to Eqs. (4–8), the position of a GW is updated.

9 $${\text{flag }}_{i,j}=\left\{\begin{array}{c}1{ X}_{i,j}>0.5\\ 0 \, \, \, \, \, \, {\text{otherwise}}\end{array}\right.$$

where ${{\varvec{X}}}_{{\varvec{i}},{\varvec{j}}}$ Designates the ${\varvec{i}}{\varvec{t}}{\varvec{h}}$ GW's ${\varvec{j}}{\varvec{t}}{\varvec{h}}$ position.

## Related work

Numerous studies have explored diverse machine learning (ML) algorithms for breast cancer (BC) diagnosis, revealing a trend toward integrating metaheuristic algorithms with convolutional neural networks (CNNs) to enhance medical image classification and analysis. Research by^33^ utilized hybridized sine cosine algorithms with CNN dropout regularization. At the same time^34^, applied advanced meta-heuristics with CNNs for efficient COVID-19 X-ray chest image classification, illustrating the potential of these approaches to improve diagnostic accuracy. Further^35^, investigation into nature-inspired metaheuristic optimized CNN models for breast cancer image analysis, along with^36^ study on glioma brain tumor grade classification using CNNs adjusted by modified firefly algorithms and^37^ innovative lung cancer detection method combining CNN-based and feature-based classifiers with metaheuristics, all highlight the efficacy of these advanced computational techniques in enhancing medical image processing and disease diagnosis. Connecting these developments to breast cancer research^38^, presents a genetically optimized neural network (GONN) specifically for BC classification, employing a genetic algorithm (GA) to refine the neural network's architecture, achieving a noteworthy accuracy rate of 97.73 percent. In^39^, a genetic algorithm is utilized for feature selection, leading to an accuracy of 95.8 percent when supplying optimized features to a support vector machine (SVM) classifier. These instances underscore the significant impact of integrating metaheuristic algorithms with ML techniques on the accuracy and efficiency of cancer diagnosis.

The prognosis of BC can be enhanced through the use of GA. By introducing a tribe competition-based GA (TCb-GA) and GA for online gradient boosting (GAOGB)for feature selection, and a naïve Bayes approximation-rule-based fuzzy BC classifier achieved an accuracy of 98.32,94.28, and 95.75 percent, respectively^40–42^. Accuracy of 96.86 and 75.05 percent were afterward achieved using a multi-objective elitism-based differential-evolution and a graph-based skill acquisition method algorithm^33,37^. Effectively categorize BC datasets tainted by impulsive noise^45^, present a multilayer extreme learning machine method based on full correntropy. Moreover, a sparse pseudoinverse incremental-ELM, likelihood-fuzzy analysis, and a two-stage BC classification using association rules with SVM for classification and reducing the number of features are proposed and achieve an accuracy of 95.26, 97.28, and 98 percent, respectively^46–48^. Resampling, discretization, and the elimination of missing values are all part of the preprocessing method used in^46^, after which three classifiers, Sequential-Minimal-Optimization, Naive Bayes, along with J48, are used for the classification of BC with an average accuracy of 97.5,98.73 and 98 percent respectively.

An SVM-RBF kernel and AdaBoost algorithm classifiers hybridized with nature-inspired algorithms utilize the maximum likelihood principle to improve classification stability. The hybridization of SVM-RBF with Particle Swarm Optimization (PSO), GA, and Ant Colony Optimization (ACO) was applied to the BC dataset. It achieved an accuracy of 96 (AdaBoost), 97.37(PSO), 97.19(GA), 95.96(ACO) using 10-CV^49,50^. In addition, K-SVM, a system that combines support vector machines with K-means, successfully identifies malignant and benign tumors with 97.38 percent accuracy^51^. Using MIAS, WDBC, and WBCD, among others, PSO has been applied to these datasets^52^.To improve the feature subset and kernel bandwidth for BC diagnosis, a kernel density estimation-PSO (KDE) technique is proposed in^53^ with an accuracy of 97.21 percent. An AISL and a select and test oncology diagnostic system (STONCODIAG) approach were proposed by ^54,55^ to improve the accuracy of 98.3, sensitivity of 94.3 and 81, and specificity of 99.6 and 100 percent, respectively.

Improving ANN performance while reducing misclassification costs is the goal of the LS-SOED method presented by^56^. Furthermore, LR for feature selection, along with the Data Handling Group Method and a smooth group L1/2 regularization technique for finding and eliminating redundant nodes in the input of feedforward NNs, is presented by^57,58^ with accuracy achieved by GMDH-NN 99.4 and precision achieved by GLSGL½ was 92.94 and 91.04 percent respectively. BC detection using^59^ offers a fuzzy interference system based on an adaptive network and a DT, ML, with average CAs of 96% and 93.7%, respectively. Hybrid ML models have been developed in recent years to address a wide range of problems using a wide range of meta-heuristic optimization approaches, such as Biogeography-Based Optimization (BBO), Grey Wolf Optimizer (GWO), Particle Swarm Optimization (PSO), Sine Cosine Algorithm (SCA), Cheetah Optimization Algorithm (ChOA), Salp Swarm Algorithm (SSA), Whale Optimization Algorithm (WOA), Adaptive Gradient Particle Swarm Optimization(AGPSO), as well as Dragonfly Algorithm (DA). Although the No Free Lunch (NFL) theorem claims that the no metaheuristic algorithm is commonly superior to any other approach, some algorithms based on metaheuristics are more effective than others when applied to specific optimization problems.

A CAD method is also introduced for splice site prediction, achieving impressive accuracies of 95.20% and 97.50% for donor and acceptor sites^60^. A hybrid convolutional neural network (CNN) and vision transformer-based framework excel in surveillance video anomaly detection, demonstrating high AUC values across benchmark datasets^61^. The Vision Transformer Anomaly Recognition (ViT-ARN) framework significantly advances intelligent city surveillance by proficiently detecting and interpreting anomalies, outperforming alternative approaches with substantial accuracy improvements^62^. This collective progress underscores the adaptability and effectiveness of customized machine-learning solutions in addressing diverse challenges.

**Figure 3:**
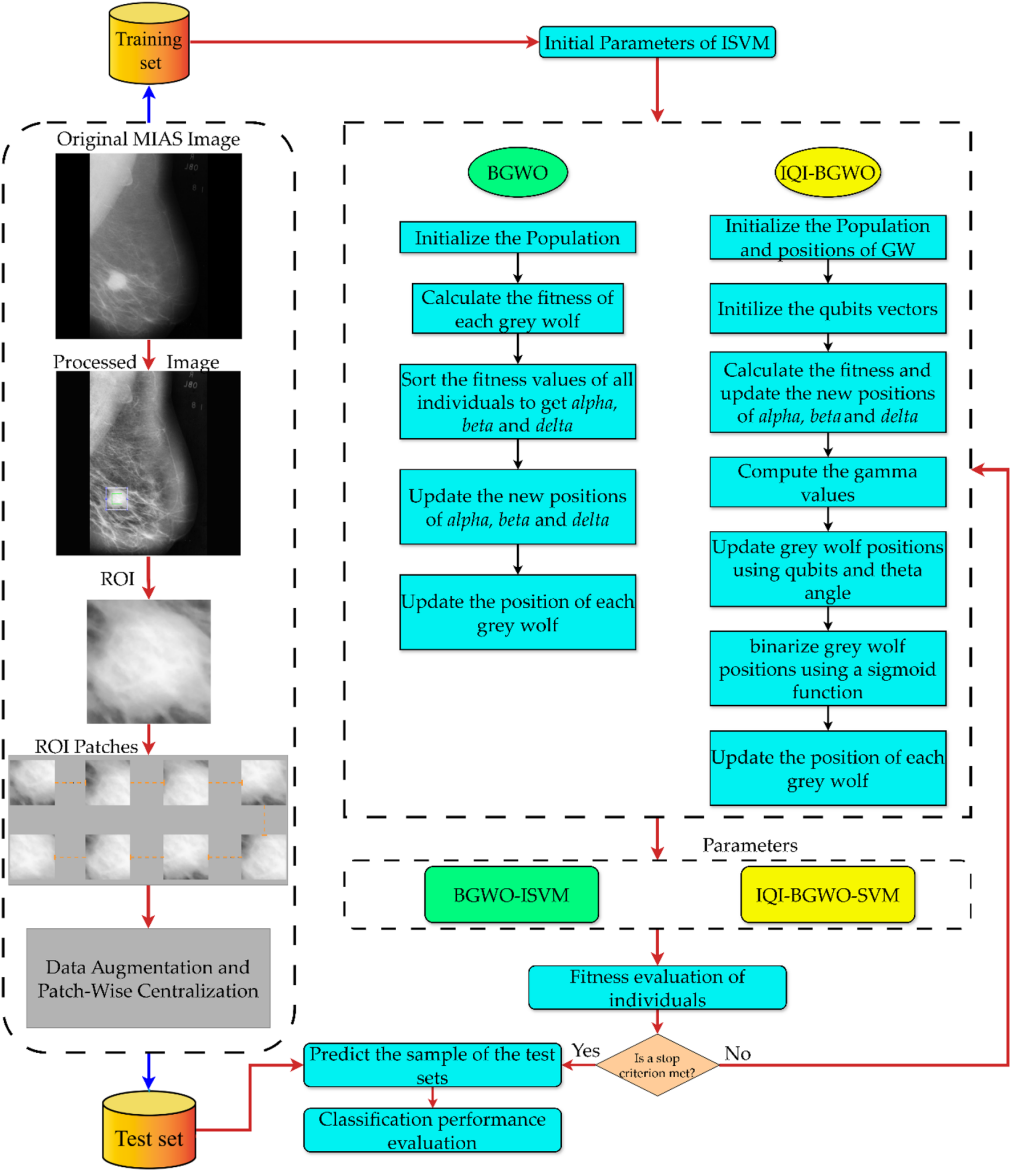
The framework of the proposed methodology.

Thus, this study intends to investigate the feasibility of using the IQI-BGWO-SVM framework to categorize mammographic images within the MIAS dataset. MIAS begins with basic image processing to eliminate background noise and improve quality. Then, regions of interest (ROI) from the benign and malignant are gathered, and ROIs are randomly extracted from the Normal class. Each anomalous region within the MIAS dataset is annotated with its center coordinates, making it possible to extract a single square area centered on this position as the ROI. Since the Normal class provides no location information, the ROI is drawn randomly from the entire image and is the size specified above^63^. In addition, the Normal and Abnormal (i.e., aberrant) classes can be somewhat differentiated. Still, benign and malignant ROIs show comparable patterns but lack distinguishability. After that, this study proposes designing BGWO and IQI-BGWO models to determine patterns that are discernable between the normal and abnormal categories. This work uses BGWO and IQI-BGWO as SVM to build an accurate classifier for BC classification as malignant or benign. This study attempts to classify BC automatically and reliably as either cancerous or benign. The SVM classifier and the BGWO or IQI-BGWO algorithms are integrated. Figure 3 illustrates the overall structure of this study.

## Material and methods

### Datasets description

The Mammographic Image Analysis Society (MIAS) database is integral to the United Kingdom's National Breast Screening Program (UK NBSP). This comprehensive collection encompasses 322 mammographic images, capturing both left and right breast views from 161 individuals^64^. The dataset consists of high-resolution grey-scale images, each with dimensions of 1024 by 1024 pixels, stored in Portable Gray Map (PGM) format. The MIAS database organizes these images into three primary categories based on the nature of the findings: there are 207 normal images, 63 benign images, and 52 malignant images. Moreover, the dataset provides a detailed classification of the images according to the type of background tissue present, which includes fatty, fatty-glandular, and dense-glandular. It also delineates the images by various etiological features. These features encompass calcifications (CALC), well-defined or circumscribed masses (CIRC), spiculated masses (SPIC), masses that are miscellaneous or ill-defined (MISC), architectural distortions (ARCH), and asymmetries (ASYM).

**Figure 4:**
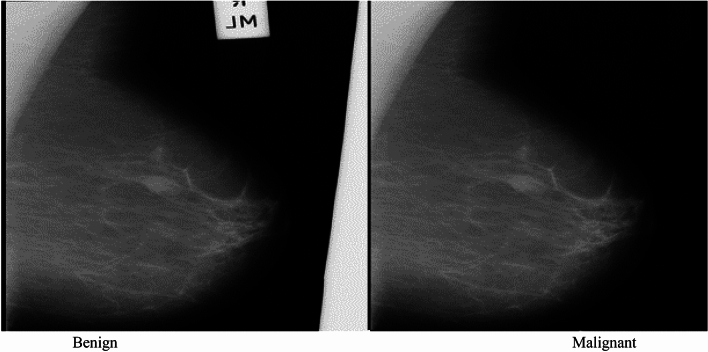
MIAS breast mammogram images.

In an illustrative example from the MIAS dataset, Fig. 4 showcases two distinct cases. The first image presents a benign tumor set against a fatty tissue background, characterized by its smooth edges and regular form, indicative of a CIRC etiology. In stark contrast, the second image shows a malignant tumor, also against a fatty background, but marked by an ASYM etiology, distinguished by its blurred boundaries and irregular shape. These comparative visual representations are crucial for elucidating the differences in how benign and malignant tumors manifest in mammographic images.

The MIAS dataset comprises standard mammographic images and includes a range of abnormal images categorized into benign and malignant types. Within this collection, there are 208 standard images and 114 abnormal images. The abnormal segment is further divided into 63 benign and 51 malignant cases. Each image in the dataset is detailed with a resolution of 1024 × 1024 pixels. For the abnormal images, specific details such as the center point of the abnormality and an estimated radius that delineates the affected area are provided, offering critical insights into the nature and extent of the abnormalities observed.

### Data preprocessing

**Figure 5:**
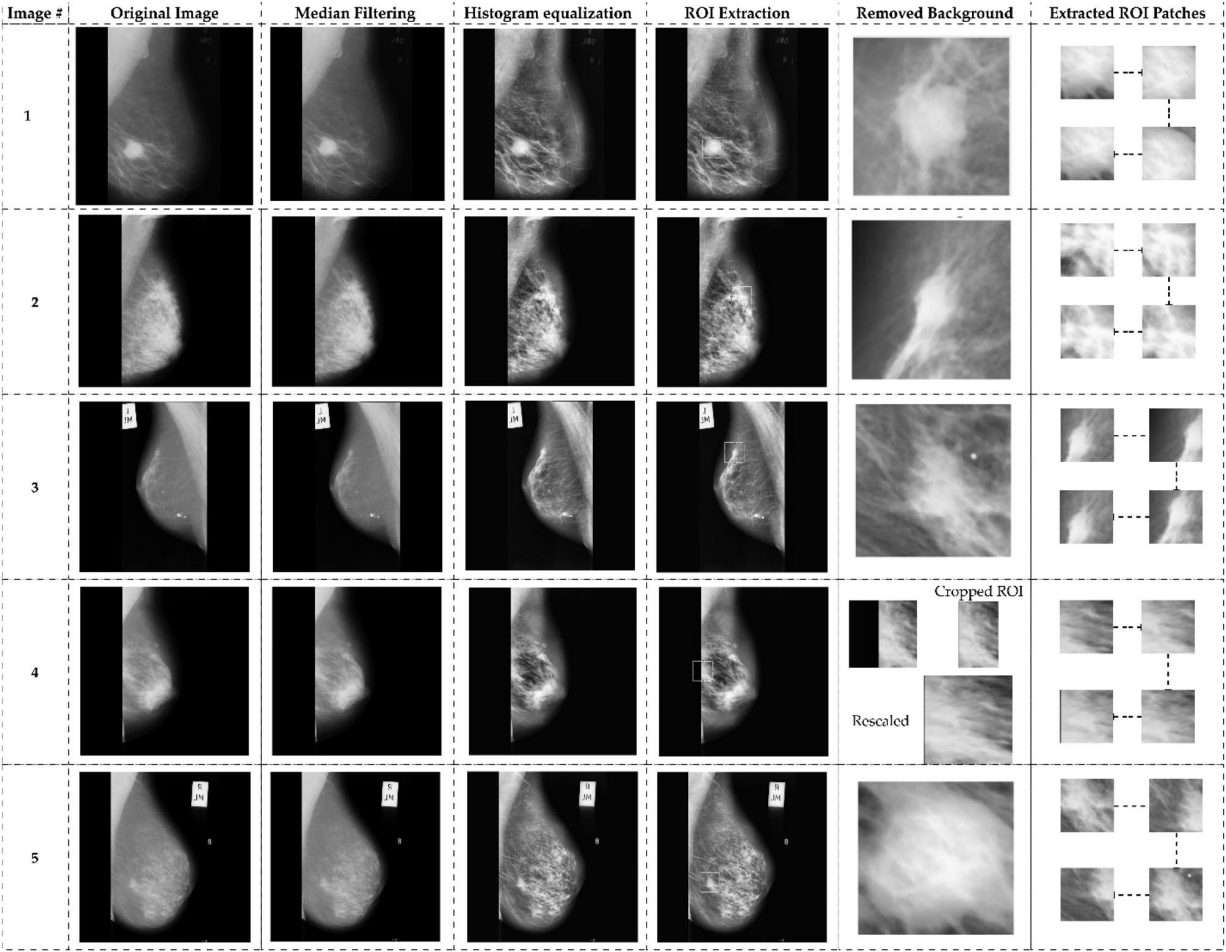
Preprocessing, ROI extraction: (**a**) original image (**b**) median filter (2) CLAHE (**d**) ROIs Extraction (**e**) ROI cropped (**f**) Extracted ROI patches to 120 × 120 pixels.

A significant amount of noise is present in the unprocessed images obtained from the MIAS dataset. Data preprocessing is required before model learning to eliminate noise and enhance image quality. Figure 5 illustrates the data preprocessing flowchart. The median filter eliminates noise, and the image is enhanced by contrast-limited adaptive histogram equalization. Following the extraction of the ROIs, a non-breast region is eliminated and rescaled to 120 × 120 pixels. After preprocessing the data, the finalized ROIs with 120 120-pixel borders encompassing 114 abnormal regions were acquired. The relevant ROIs are extracted at a randomized center inside the breast region for normal images, each measuring 120 × 120 pixels. A total of 207 normal and 119 abnormal ROIs (68 benign and 51 malignant). After obtaining ROIs from 207 normal and 119 abnormal images, indiscriminately extracted 72 × 72 pixels patches of each ROI.

### Improved quantum-inspired binary grey wolf optimization

The original Grey Wolf Optimizer (GWO) uses continuous values in the range of ^0,1^ for the positions of all wolves. In contrast, the Binary-GWO (BGWO) represents the position of each wolf as a binary value, which is calculated using a sigmoid function applied to the GW positions. To solve the unit commitment problem, a quantum-inspired BGWO was introduced^18^, which proposed an IQI-BGWO to address the selection of feature problems. In IQI-BGWO, the position of each wolf is binary and updated based on a particular qubit vector along with a quantum rotational gate, each wolf having its qubit as well as rotation gate. The $y\_gate\left( \theta \right)$ Eq. (3) is used for this purpose. While the original GWO updates each wolf position using equations A as well as C, in IQI-BGWO, the location update is dependent on the qubit corresponding to each wolf and the angle θ of each wolf, which is updated based on two probabilistic random γ, ζ values, as demonstrated in the subsequent equations.

10 $$\left( \theta \right)_{\alpha } \left( {t + 1} \right) = \left( {\upzeta } \right)_{\alpha } \left( {\upgamma } \right)_{\alpha } \sum \left( {\overset{\lower0.5em\hbox{$\smash{\scriptscriptstyle\rightharpoonup}$}}{{\left( {\text{X}} \right)_{\alpha } }} \cdot \left( t \right) - \overset{\lower0.5em\hbox{$\smash{\scriptscriptstyle\rightharpoonup}$}}{\left( X \right)} \cdot \left( t \right)} \right) \cdot 2\pi$$

11 $$\left( \theta \right)_{\beta } \left( {t + 1} \right) = \left( {\upzeta } \right)_{\beta } \left( {\upgamma } \right)_{\beta } \sum \left( {\overset{\lower0.5em\hbox{$\smash{\scriptscriptstyle\rightharpoonup}$}}{{\left( {\text{X}} \right)_{\beta } }} .\left( t \right) - \overset{\lower0.5em\hbox{$\smash{\scriptscriptstyle\rightharpoonup}$}}{\left( X \right)} \cdot \left( t \right)} \right) \cdot 2\pi$$

12 $$\left( \theta \right)_{\delta } \left( {t + 1} \right) = \left( {\upzeta } \right)_{\delta } \left( {\upgamma } \right)_{\delta } \sum \left( {\overset{\lower0.5em\hbox{$\smash{\scriptscriptstyle\rightharpoonup}$}}{{\left( {\text{X}} \right)_{\delta } }} \cdot \left( t \right) - \overset{\lower0.5em\hbox{$\smash{\scriptscriptstyle\rightharpoonup}$}}{\left( X \right)} \cdot \left( t \right)} \right) \cdot 2\pi$$

13 $${\left(\upzeta \right)}_{\alpha }={\left(\uplambda \right)}_{1}.\pi$$

14 $${\left(\upzeta \right)}_{\beta }={\left(\uplambda \right)}_{2}.\pi$$

15 $${\left(\upzeta \right)}_{\delta }={\left(\uplambda \right)}_{3}.\pi$$

where θ represents the angle for the quantum rotation gate used in updating the position of each wolf, *α, β,* and *δ* denotes the leading wolves in the hierarchy, guiding the search process. ζ and γ are probabilistic random values influencing the rotation angle θ for each wolf, reflecting the stochastic nature of the algorithm.${\left(\uplambda \right)}_{1},{\left(\uplambda \right)}_{2}$ and ${\left(\uplambda \right)}_{3}$​ are random values assigned to each of the leading wolves, affecting the magnitude of ζ for each wolf.

In the context of FFEs, the IQI-BGWO modifies the computation by integrating quantum principles, potentially altering the number of FFEs compared to the baseline GWO. The complexity of FFEs is higher in IQI-BGWO due to the additional quantum computations. Specifically, the fitness evaluation in IQI-BGWO involves quantum state adjustments and rotation, which adds layers to the computational process. Compared to the baseline GWO, where FFEs are direct evaluations of the fitness function, IQI-BGWO requires more computational steps, including the quantum rotation and state update processes.

Random values λ1, λ2, and λ3 are assigned to α, β, as well as δ wolves, and ζα represents the theta magnitude for the α wolf. The corresponding rotation angle is used to rotate each wolf's qubit vector, denoted as Q, according to Eqs. (16), (17), and (18).

16 $$\left( Q \right)_{\alpha } \left( {t + 1} \right) = \left( R \right)_{\alpha } \cdot \left( {\left( \theta \right)_{\alpha } \cdot \left( {t + 1} \right)} \right) \cdot \left( Q \right)_{\alpha } \left( t \right)$$

17 $$\left( Q \right)_{\beta } \left( {t + 1} \right) = \left( R \right)_{\beta } \cdot \left( {\left( \theta \right)_{\beta } \cdot \left( {t + 1} \right)} \right) \cdot \left( Q \right)_{\beta } \left( t \right)$$

18 $$\left( Q \right)_{\delta } \left( {t + 1} \right) = \left( R \right)_{\delta } \cdot \left( {\left( \theta \right)_{\delta } \cdot \left( {t + 1} \right)} \right) \cdot \left( Q \right)_{\delta } \left( t \right)$$

where Q is a quantum state vector that forms a single qubit. R denotes the rotation operation applied to the qubit vector Q of each wolf, with ${\left(Q\right)}_{\alpha },{\left(Q\right)}_{\beta },$ and ${\left(Q\right)}_{\delta }$ Representing the qubit states of the respective wolves.

19 $$\left|{\left(Q\right)}_{\alpha }\rangle ={\left(x\right)}_{\alpha }\right|0\rangle +{\left(y\right)}_{\alpha }|1\rangle$$

20 $$\left|{\left(Q\right)}_{\beta }\rangle ={\left(x\right)}_{\beta }\right|0\rangle +{\left(y\right)}_{\beta }|1\rangle$$

21 $$\left|{\left(Q\right)}_{\delta }\rangle ={\left(x\right)}_{\delta }\right|0\rangle +{\left(y\right)}_{\delta }|1\rangle$$

where* x* and *y* are the coefficients in the superposition of the qubit states, indicating the probability amplitudes for the quantum states ∣0⟩ and ∣1⟩.The initial values of ${\left(x\right)}_{\alpha }$, ${\left(y\right)}_{\alpha }$, ${\left(x\right)}_{\beta }$, ${\left(y\right)}_{\beta }$, ${\left(x\right)}_{\delta }$, and ${\left(y\right)}_{\delta }$ Are set to 1/2. The wolves' locations are updated based on the probability of the qubit vector being in state |1〉, as follows:

22 $${\left(X\right)}_{\alpha }\left(t+1\right)={\left(X\right)}_{\alpha }\left(t\right).({y}_{\alpha }^{2})(t+1)$$

23 $${\left(X\right)}_{\beta }\left(t+1\right)={\left(X\right)}_{\beta }\left(t\right).({y}_{\beta }^{2})(t+1)$$

24 $${\left(X\right)}_{\delta }\left(t+1\right)={\left(X\right)}_{\delta }\left(t\right).({y}_{\delta }^{2})(t+1)$$

Using a straightforward thresholding operation, the probabilistic values associated with wolves' positions are converted to binary values that are as follows:

25 $$\overset{\lower0.5em\hbox{$\smash{\scriptscriptstyle\rightharpoonup}$}}{{\left( {X_{\alpha } } \right)}} .\left( {t + 1} \right) = \left\{ {\begin{array}{*{20}c} {1\; if\; \overset{\lower0.5em\hbox{$\smash{\scriptscriptstyle\rightharpoonup}$}}{{\left( {X_{\alpha } } \right)}} .\left( {t + 1} \right) \ge y_{\alpha }^{2} \left( {t + 1} \right)} \\ {0 \;otherwise} \\ \end{array} } \right.$$

The first step is to threshold the values of the wolves' positions to obtain binary values for each feature. As described previously, the threshold is determined based on the qubit probability of state |1〉 for each wolf. The second step is to perform a majority voting scheme for the binary values of each feature among the solutions provided by the α, β, and δ wolves. If most wolves have a binary value of 1 for a particular feature, the final binary value for that feature is set to 1; otherwise, it is set to 0. This procedure results in a binary feature vector $$\overset{\lower0.5em\hbox{$\smash{\scriptscriptstyle\rightharpoonup}$}}{\left( X \right)}$$ That represents the selected features for the problem at hand.

1- Use an equation based on the sigmoid function (26) on $$\overset{\lower0.5em\hbox{$\smash{\scriptscriptstyle\rightharpoonup}$}}{\left( X \right)}$$ for F $$\overset{\lower0.5em\hbox{$\smash{\scriptscriptstyle\rightharpoonup}$}}{\left( X \right)}$$

2- F $$\overset{\lower0.5em\hbox{$\smash{\scriptscriptstyle\rightharpoonup}$}}{\left( X \right)}$$ is compared to a randomized value such that λ.

$${\text{a}}.{ }\overset{\lower0.5em\hbox{$\smash{\scriptscriptstyle\rightharpoonup}$}}{\left( X \right)} = \left\{ {\begin{array}{*{20}c} {1\; F\overset{\lower0.5em\hbox{$\smash{\scriptscriptstyle\rightharpoonup}$}}{\left( X \right)} \ge s } \\ {0\; F\overset{\lower0.5em\hbox{$\smash{\scriptscriptstyle\rightharpoonup}$}}{\left( X \right)} < s} \\ \end{array} } \right.$$

Where s takes on a value from 0 to 1, a sigmoid-function has the following form:

26 $$sigm\left( p \right) = \frac{1}{{1 + e^{{\left( { - p} \right)}} }}$$

p indicates the value's position and takes values ranging from [0 to 1]. The pseudocode of the IQI-BGWO is presented in Table 2:

**Table 2:** Pseudocode of the IQI-BGWO.

Given variables
`N_iter`: Total iterations for the optimization process
`n`: Number of wolves, representing feature quantity
*1. Quantum Population Initialization*
For index ` i' starting at 1 to `n`:
Assign a quantum state to `wolf_position[i]`
Initialize control parameters `xi` and `yi` as 0.5
Conclude initialization with the `wolf_position` output
*2. Fitness Evaluation of Wolf*
Define and return `EvaluateFitness(wolf)`
*3. Quantum State Adjustment*
Update quantum rotation angles using specified formulas (32–37)
Refresh states of alpha (`α`), beta (`β`), and delta (`δ`) wolves via quantum calculations
*4. Quantum Position Refinement*
Apply quantum rotation on `wolf`'s current position following formulas (38–40)
Update `wolf`'s position with the new calculations from formulas (44–46)
*5. Optimization Execution*
Begin with `wolf_positions` populated by a quantum population of size `n`
Iterate from 1 to `N_iter`:
Execute `UpdateParameters()` and for each `wolf` in `wolf_positions`:
Evaluate and record the fitness of `wolf` using `EvaluateFitness(wolf)`
Refine `wolf`'s quantum position
Complete the iteration cycle
Apply a defined thresholding method (Eq. 25) for feature selection
Identify and return the feature set from `wolf_positions` with the top fitness values

### Improved quantum-inspired binary grey wolf optimization for feature selection

Feature selection serves as a critical step in the realm of machine learning. Its primary role is to trim down the dimensionality of the dataset by selectively retaining features that contribute the most to learning accuracy. This becomes increasingly vital when working with large-scale datasets or tackling machine learning tasks, where computational efficiency and model performance are paramount. This study employs the IQI-BGWO to select features. This algorithm is designed to optimize the subset of features used for training the model, aiming to balance reducing dimensionality and improving classification accuracy. To validate the effectiveness of IQI-BGWO in feature selection, utilize the optimized ISVM classifier as a machine learning model. ISVM is a supervised learning algorithm that uses labeled data to generate a predictive model. It is well-suited for evaluating the quality of the selected features because it is sensitive to irrelevant or redundant features. The primary evaluation criterion focuses on achieving the most minor possible feature set while minimizing the error rate. This dual-objective assessment reduces computational complexity and sustains high model performance. The assessment criteria are encapsulated in a fitness function, framed as a minimization problem, and represented by Eq. 27.

27 $$fitness=q{P}_{r}\left(M\right)+e\frac{|R|}{|C|}$$

When reducing the number of features and improving classification accuracy, use the constants e = 1-q and q ϵ ^0,1^ to use e = 0.01 in this study. R is the length of the subset of features chosen for further analysis, and C is the total number of features in $q{P}_{r}\left(M\right)$.An in-depth analysis of ten individual experiments conducted using the IQI-BGWO. Each experiment is distinctly numbered for easy tracking, running from 1 to 10. On average, the algorithm achieved a fitness value of approximately 0.0558, though individual trials showed results ranging from as low as 0.0150 to as high as 0.1100. This suggests that while IQI-BGWO is generally effective, its efficiency can fluctuate depending on the specific dataset and initial conditions used in each experiment. In the context of feature elimination, the number of discarded features oscillates slightly between 7 and 9, with an average close to 7.9. This minor fluctuation underscores the algorithm's ability to adapt its feature selection based on the unique attributes of the dataset. Lastly, the iteration count needed to reach the optimal solution varies noticeably, spanning from a mere 8 iterations to an extensive 58, with the average hovering around 26. This variation hints at the algorithm's efficiency but suggests that more complex problems might require additional iterations to reach the optimum.

A meticulous account of the performance of the Improved Quantum-Inspired Binary Grey Wolf Optimizer (IQI-BGWO) algorithm over ten separate trials. Each trial is uniquely numbered under the "Trial No." column for straightforward reference. The general hyperparameters and those specific to the IQI-BGWO algorithm. General hyperparameters include CV, the cross-validation set consistently at 10 for all trials; I, the total number of iterations, fixed at 100; and P.S, the population size, set at 8 across all trials. The objective function to be minimized, denoted by F, is uniformly represented as n ∗ , and its domain, D, is confined to the range ^0,1^.

Additionally, two weighting parameters for the fitness function, α and β, are set at 0.99 and 0.01, respectively. The "Optimal Iteration" column specifies the iteration count at which each trial yielded its best fitness value, which is then reported under the "Best Fitness Value" column. Special to the IQI-BGWO are parameters like θα​, which indicates the θ value for the α wolf in each trial, and Qα​, the qubit vector specific to the α wolf. Finally, s represents the threshold the sigmoid function uses for converting probabilistic values to binary. This comprehensive table is a robust tool for evaluating the algorithm's efficacy and understanding its behavior across different trials.

### Improved SVM-RBF

The improved SVM-RBF is a versatile aggregation technique suitable for regression and classification tasks. Unlike conventional statistical-based parametric classification methods, the ISVM-RBF is non-parametric. While SVM is one of the most widely used non-parametric ML algorithms, its performance deteriorates when dealing with large amounts of data. Therefore, the new ISVM-RBF is designed to enhance the efficiency and accuracy of change detection without any assumptions about the data distribution. To handle nonlinear data, the nonlinear ISVM-RBF leverages kernel functions to reduce computational complexity, a technique known as the kernel trick. Popular kernel functions include the polynomial kernel and Gaussian kernel.

In the case of non-linearly separable data, SVM-RBF uses a nonlinear mapping function to convert the input parameters to a higher dimensional space for features., where a hyperplane is constructed to achieve the best classification. This process is recognized as a kernel trick, allowing for efficient computation of the inner product between two vectors without actually computing the transformation. The SVM-RBF can use various kernel functions, but the polynomial and Gaussian kernels are the most commonly used. In this study, the authors focused on the radial basis function (RBF) kernel, a type of Gaussian kernel that has been enhanced for better performance.

The improved ISVM-RBF incorporates two parameters, λ and σ. The parameter σ is used in the execution of the function, and λ is crucial as it determines the compromise between the predicted function along with the minimum fitting error. Therefore, the improved SVM-RBF can be computed as follows:

28 $$ISVM-RBF=\forall \omega \left(a,{a}_{i}\right)={\text{exp}}\left(-\frac{1}{{\sigma }^{2}}{\left|\left|a,{a}_{i}\right|\right|}^{2}\right)\times \lambda$$

The first condition for the non-linearity characteristics of SVM-RBF is that it must be symmetric, and the second condition is that it should be capable of ensuring space identification with the problems in the real world, which is the pairwise integrating potential. Equations 28 and 29 establish these two conditions, where ∀ω*($a,{a}_{i}$) represents the improved SVM-RBF attributes, as well as the variant function, is represented by ∀ω .

29 $$\forall \omega \left(\left(a,p\right)=\left(\varphi \left(a\right).\varphi \left(p\right)\right)\right)$$

30 $$\forall \omega \left(a,p\right)-\left\{\varphi \left(a\right).\varphi \left(p\right)\right\}=\left\{\left(a\right).\varphi \left(p\right)-\forall \omega \left(a,p\right)\right\}$$

The one verses all technique can integrate binary classifiers with SVM-RBF. In the context of a K-classification issue, the one verses all approach generates a single binary classifier for each class. In this method, all samples of a particular class have y = 1, and all samples of the remaining (k-1) classes have y = 0. Therefore, there will be k-binary classifiers in total. All k binary classifiers will be executed to classify new data x, and it will be classified into the class of i, providing the most significant probability as well as classification result.

The choice of the SVM-RBF as the classifier in this research is grounded in its exceptional ability to manage nonlinear data through the kernel trick. This approach is crucial for datasets where the relationship between features is complex and not linearly separable. The kernel trick allows the SVM-RBF to project data into a higher-dimensional space, facilitating a more nuanced and effective classification boundary than linear models could achieve. This capability is particularly advantageous for complex classification tasks where the intricacies of data relationships need to be accurately captured.

The SVM-RBF framework is also valued for its robustness in handling high-dimensional data spaces. It maintains performance even when the dataset features are large compared to the number of samples, a scenario where many models tend to overfit. Overfitting compromises the model's ability to generalize to new data, but the SVM-RBF's structural design inherently avoids this pitfall, thus ensuring more reliable predictions.

Moreover, the improved version of SVM-RBF, or ISVM-RBF, introduces enhancements that address some of the conventional SVM limitations, such as scalability and computational efficiency. These improvements are particularly relevant when dealing with large datasets. By fine-tuning the model parameters, λ and σ, the ISVM-RBF achieves a balance that enhances the model's performance and computational efficiency. This balance is crucial for practical applications where accuracy and processing speed are essential.

While other classifiers like XGBoost, AdaBoost, and Random Forest are effective in various scenarios, their appropriateness depends heavily on the specific characteristics of the problem and the data at hand. For instance, while Random Forest is adept at handling datasets with many features and can deal with nonlinear relationships, it may not provide the same level of performance as SVM-RBF in situations where the separation margin between classes in the feature space is minimal. Therefore, the selection of ISVM-RBF for this study was strategic, aimed at leveraging its specific strengths in handling the unique challenges posed by the dataset. This included its proficiency in dealing with nonlinear separability and high-dimensional spaces and its ability to avoid overfitting, thus ensuring that the model remains effective and reliable when applied to new and unseen data.

### ISVM-RBF optimization

**Figure 6:**
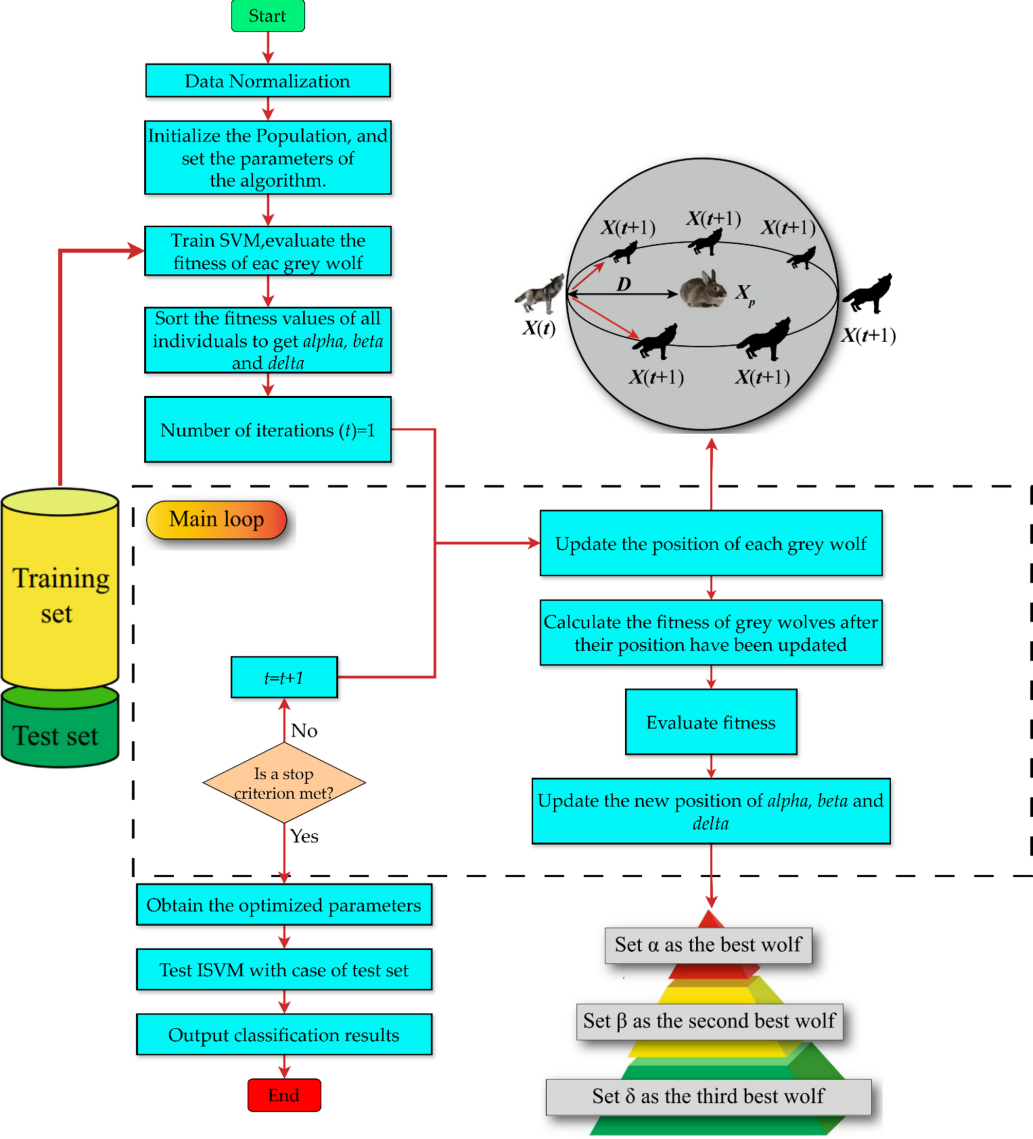
Flow chart of IQI-BGWO-SVM.

The optimal values of parameters are crucial for achieving a superior classification rate while training the SVM classifier. Optimization algorithms such as IQI-BGWO and BGWO are employed with the SVM classifier to obtain these ideal values. This results in the optimal classification accuracy for the classifier. The proposed system is described in Fig. 6. Once the optimal parameters of the SVM are obtained, the dataset is trained to obtain the learning model, which is subsequently utilized to anticipate the test data and obtain the highest possible classification accuracy. Instructions for implementing the recommended optimal SVM model are as follows:

1. First, generate a random population of GW's. Optimal ISVM performance depends on balancing two parameters; therefore, data on every individual is stored in a two-dimensional array. The next step is to determine the fundamental IQI-BGWO parameters. Train the ISVM and assess the fitness of each search agent.
2. The IQI-BGWO-SVM's fitness function is created following its classification accuracy throughout cross-validation. This study takes advantage of the K-fold CV method, which can assess ISVM's generalizability accurately. This work uses cross-validation at three different levels: 5, 10, and 15 CV, along with the fitness function developed based on the performance of the training set in the CV.

When reducing the number of features and improving classification accuracy, use the constants e = 1-q and q ϵ ^0,1^ to use e = 0.01 in this study. R is the length of the subset of features chosen for further analysis, and C is the total number of features in $q{P}_{r}\left(M\right)$.

3. In step 3, when an initial population has been generated using data set samples as input to the model, the fitness of every one of them is determined using the fitness function. Their fitness levels are ranked from highest to lowest to determine which three grey wolves have the greatest hunting skills. These wolves are then given the names α, β, and δ.
4. The location updates of all the grey wolves will be coordinated when the initial values of α, β, and δ have been chosen. This results in the formation of a new population of grey wolves in which the roles of the individuals have shifted. After that, an assessment and computation of every individual's fitness level is carried out. The population is broken up into α, β, δ, and ω accordingly. The preceding process will repeat indefinitely if the maximum possible number of iterations has not been attained.
5. After iteration, the model will output the optimal solution, substituting it into ISVM to create an optimal classifier. The effectiveness of the hybrid classification framework is evaluated next using test set samples drawn from the whole dataset.

### Performance metrics

To assess the classification performance of the IQI-BGWO–SVM and BGWO–SVM models, this study employs a set of established performance metrics, which are pivotal in machine learning and statistical analysis for evaluating the efficacy of classification models^65–67^. The chosen metrics are Accuracy, Specificity, Sensitivity, Error Rate, and Matthew's Correlation Coefficient (MCC), each serving a distinct purpose in quantifying model performance.

Accuracy (ACC) represents the proportion of correctly classified samples (both true positives and true negatives) to the overall sample count. It is calculated as:

31 $$ACC=\frac{{\text{TP}}+{\text{TN}}}{{\text{TP}}+{\text{TN}}+{\text{FP}}+{\text{FN}}}\times 100\mathrm{\%}$$

where (TP) is true positives, (TN) is true negatives, (FP) is false positives, and (FN) is false negatives.

Specificity (SPC) measures the proportion of actual negatives correctly identified as such (true negatives) and is vital for assessing the model's ability to identify negative cases. It is computed as:

32 $$SPC=\frac{{\text{TN}}}{{\text{TN}}+{\text{FP}}}\times 100\mathrm{\%}$$

Sensitivity (SEN)indicates the model's ability to identify positive cases correctly. It is the proportion of actual positive samples that are correctly classified as positive:

33 $$SEN=\frac{{\text{TP}}}{{\text{TP}}+{\text{FN}}}\times 100\mathrm{\%}$$

Error Rate (E.R) calculates the proportion of all incorrect predictions (both false positives and false negatives) to total predictions, giving an overall measure of the misclassification:

34 $${\text{E}}.{\text{R}}=1-0.5\times \frac{\mathrm{SEN value}+\mathrm{SPC values}}{100}\times 100\mathrm{\%}$$

Matthew's Correlation Coefficient (MCC) provides a balanced measure that considers true and false positives and negatives, suitable for imbalanced datasets. It is defined as:

35 $$MCC=\frac{{\text{TP}}\times {\text{TN}}-{\text{FP}}\times {\text{FN}}}{\sqrt{(TP+FP)(TP+FN)(TN+FP)(TN+FN)}}\times 100\mathrm{\%}$$

These metrics are selected based on their ability to view the model's performance comprehensively. Accuracy offers an overall effectiveness rate, while Specificity and Sensitivity give insights into the model's ability to identify each class correctly. The Error Rate provides a direct measure of the model's misclassification. MCC offers a balanced metric considering all aspects of the confusion matrix, making it particularly useful for evaluating models on imbalanced datasets.

In addition to Accuracy, this study includes Specificity, Sensitivity, Error Rate, and MCC to ensure a holistic evaluation of the classification models, accounting for various aspects of performance that single metrics like Accuracy cannot fully capture. These metrics collectively enable a detailed assessment of the models' ability to effectively classify and distinguish between different classes, considering both the positive and negative instances.

## Experimental results

The proposed optimized classification techniques IQI-BGWO-ISVM and BGWO-ISVM) were implemented using MATLAB with GPU acceleration. The system configuration employed an Intel Core i7 8th generation processor with 32 GB RAM. The MIAS dataset is used to evaluate the performance of the proposed approaches. The performance of the IQI-BGWO algorithm was systematically evaluated through experiments, detailed in Table 3. The evaluation framework involved ten-fold cross-validation, limiting the iterations to 200 and setting the population size to 10. Additionally, unique IQI-BGWO parameters were considered, such as θα (representing the rotational angle for the alpha wolf) and Qα (denoting the qubit vector for the alpha wolf). This analysis aimed to pinpoint the iteration achieving the best fitness alongside other critical performance metrics.

**Table 3:** Performance and Hyperparameters metrics of IQI-BGWO.

No						θα	Optimal Iteration	Alpha Qubit Vector	Best fitness values	s
1	Cross-Validation 5,10,15	Population size 10	Objective function n*	Domain [0,1]	Weighting parameters for the fitness function α .99 and β 0.01	0.25	15	[0.55,0.55]	0.0210	0.4
2						0.30	30	[0.60,0.60]	0.0250	0.3
3						0.35	45	[065,0.65]	0.0310	0.3
4						0.40	60	[0.70,0.70]	0.0180	0.5
5						0.45	75	[0.75,0.75]	0.0290	0.4
6						0.50	90	[0.80,0.80]	0.0340	0.2
7						0.55	105	[0.85,0.85]	0.0270	0.5
8						0.60	120	[0.90,0.90]	0.0220	0.4
9						0.55	135	[0.95,0.95]	0.0260	0.3
10						0.70	150	[1.00,1.00]	0.0300	0.6
Average						0.45	82.5	Varried	0.0273	0.4

### Evaluating IQI-GBGWO relative to bio-inspired optimization techniques

**Figure 7:**
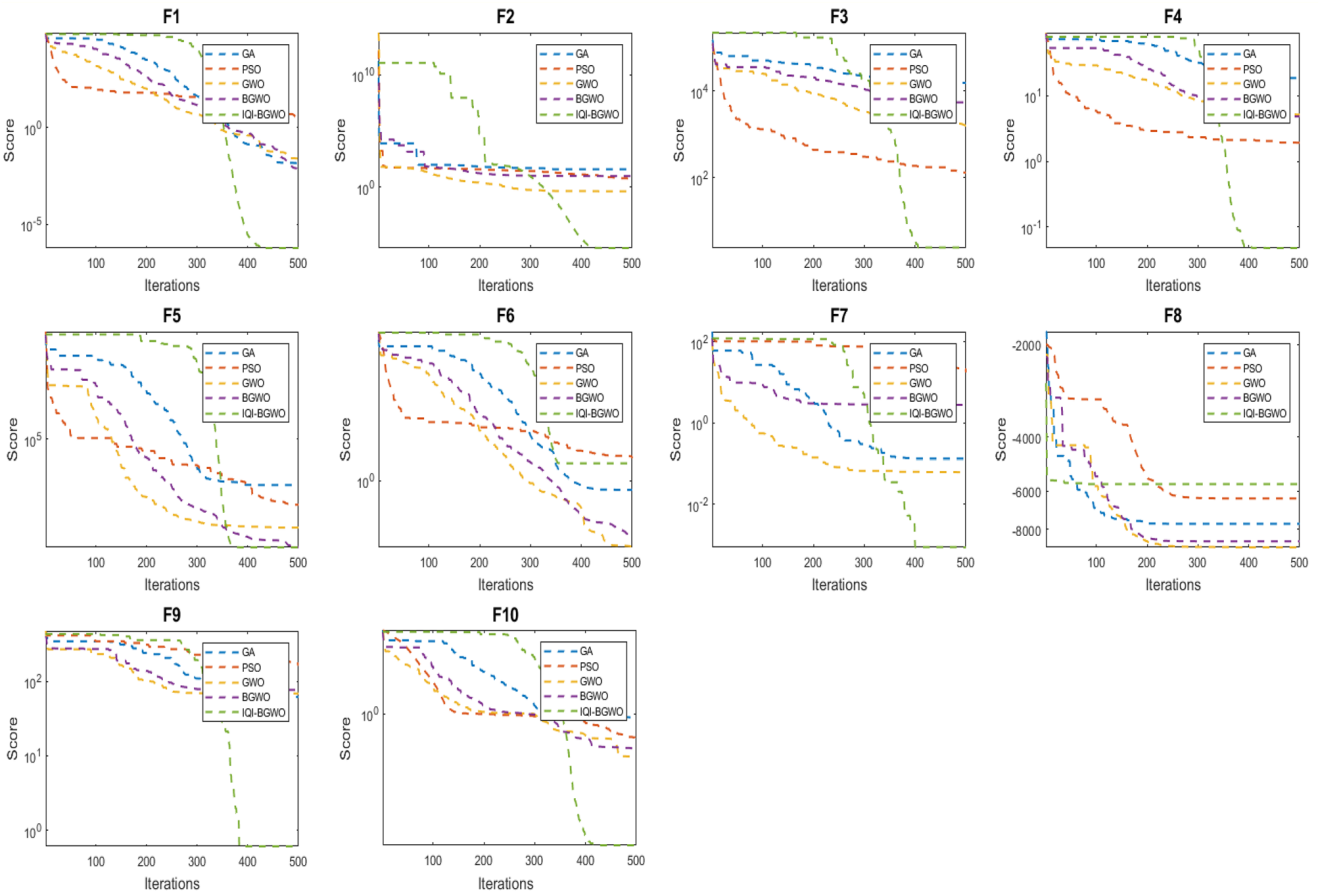
Analyzing the convergence patterns of IQI-BGWO on standard test functions.

This study compares the effectiveness of the Improved Quantum-Inspired Binary Grey Wolf Optimizer (IQI-BGWO) with traditional bio-inspired optimization algorithms, including the Grey Wolf Optimizer (GWO), Binary GWO, Particle Swarm Optimization (PSO), and Genetic Algorithm (GA). The comparison is conducted through testing on ten benchmark functions, divided into five unimodal functions (Sphere (F1), Schwefel 2.22 (F2), Schwefel 1.2 (F3), Schwefel 2.21 (F4), Generalized Rosenbrock (F5)) and five multimodal functions (Generalized Schwefel (F6), Rastrigin (F7), Ackley (F8), Griewank (F9), Generalized Penalized (F10)). Each algorithm's configuration adhered to the specifications described in their respective foundational studies. This study's findings, summarized in Table 4, reveal that the IQI-BGWO consistently delivers robust performances across various tests, often surpassing the conventional algorithms in numerous benchmarks. Although GWO, BGWO, PSO, and GA demonstrated proficiency in certain areas, the IQI-BGWO was frequently more efficient or on par across most functions evaluated. Furthermore, an analysis of convergence trends, illustrated in Fig. 7, highlights the IQI-BGWO's capability to effectively balance exploration and exploitation throughout the optimization process, demonstrating its superior adaptability and efficiency from the initial to the final stages of optimization.

**Table 4:** Performance metrics of various optimization algorithms on different functions.

Function	Metric	IQI-BGWO	BGWO	GWO	PSO	GA
F1	Best	$1.8\times {10}^{-27}$	$3.2\times {10}^{-24}$	$4.7\times {10}^{-18}$	$7.0\times {10}^{0}$	$9.3\times {10}^{2}$
	Worst	$2.0\times {10}^{-27}$	$3.5\times {10}^{-24}$	$5.2\times {10}^{-18}$	$7.2\times {10}^{0}$	$9.8\times {10}^{2}$
	Mean	$1.9\times {10}^{-27}$	$3.35\times {10}^{-24}$	$5.0\times {10}^{-18}$	$7.1\times {10}^{0}$	$9.55\times {10}^{2}$
	Std	$1.0\times {10}^{-28}$	$1.5\times {10}^{-25}$	$2.5\times {10}^{-19}$	$0.1\times {10}^{0}$	$2.5\times {10}^{2}$
	Variance	$1.0\times {10}^{-56}$	$2.25\times {10}^{-50}$	$6.25\times {10}^{-38}$	$1.0\times {10}^{-2}$	$6.25\times {10}^{4}$
F2	Best	$2.1\times {10}^{-16}$	$1.4\times {10}^{-14}$	$2.3\times {10}^{0}$	$7.5\times {10}^{0}$	$1.3\times {10}^{-11}$
	Worst	$2.3\times {10}^{-16}$	$1.5\times {10}^{-14}$	$2.4\times {10}^{0}$	$7.7\times {10}^{0}$	$1.5\times {10}^{-11}$
	Mean	$2.2\times {10}^{-16}$	$1.45\times {10}^{-14}$	$2.35\times {10}^{0}$	$7.6\times {10}^{0}$	$1.4\times {10}^{-11}$
	Std	$1.0\times {10}^{-17}$	$5.0\times {10}^{-16}$	$0.05\times {10}^{0}$	$0.1\times {10}^{0}$	$1.0\times {10}^{-12}$
	Variance	$1.0\times {10}^{-34}$	$2.5\times {10}^{-31}$	$2.5\times {10}^{-3}$	$1.0\times {10}^{-2}$	$1.0\times {10}^{-24}$
F3	Best	$1.15\times {10}^{0}$	$18.0\times {10}^{0}$	$3.85\times {10}^{3}$	$4.05\times {10}^{4}$	$4.10\times {10}^{4}$
	Worst	$1.2\times {10}^{0}$	$18.5\times {10}^{0}$	$3.90\times {10}^{3}$	$4.10\times {10}^{4}$	$4.15\times {10}^{4}$
	Mean	$1.175\times {10}^{0}$	$18.25\times {10}^{0}$	$3.875\times {10}^{3}$	$4.075\times {10}^{4}$	$4.125\times {10}^{4}$
	Std	$0.025\times {10}^{0}$	$0.25\times {10}^{0}$	$25\times {10}^{0}$	$250\times {10}^{0}$	$25\times {10}^{0}$
	Variance	$6.25\times {10}^{-4}$	$6.25\times {10}^{-2}$	$625\times {10}^{0}$	$6.25\times {10}^{4}$	$625\times {10}^{0}$
F4	Best	$5.9\times {10}^{-5}$	$3.9\times {10}^{-2}$	$20.9\times {10}^{0}$	$50.5\times {10}^{0}$	$51.0\times {10}^{0}$
	Worst	$6.1\times {10}^{-5}$	$4.1\times {10}^{-2}$	$21.1\times {10}^{0}$	$50.7\times {10}^{0}$	$51.2\times {10}^{0}$
	Mean	$6.0\times {10}^{-5}$	$4.0\times {10}^{-2}$	$21.0\times {10}^{0}$	$50.6\times {10}^{0}$	$51.1\times {10}^{0}$
	Std	$1.0\times {10}^{-6}$	$1.0\times {10}^{-3}$	$0.1\times {10}^{0}$	$0.1\times {10}^{0}$	$0.1\times {10}^{0}$
	Variance	$1.0\times {10}^{-12}$	$1.0\times {10}^{-6}$	$1.0\times {10}^{-2}$	$1.0\times {10}^{-2}$	$1.0\times {10}^{-2}$
F5	Best	$27.8\times {10}^{0}$	$29.0\times {10}^{0}$	$3.9\times {10}^{3}$	$117\times {10}^{3}$	$119\times {10}^{3}$
	Worst	$28.0\times {10}^{0}$	$29.2\times {10}^{0}$	$3.92\times {10}^{3}$	$117.5\times {10}^{3}$	$119.5\times {10}^{3}$
	Mean	$27.9\times {10}^{0}$	$29.1\times {10}^{0}$	$3.91\times {10}^{3}$	$117.25\times {10}^{3}$	$119.25\times {10}^{3}$
	Std	$0.1\times {10}^{0}$	$0.1\times {10}^{0}$	$10\times {10}^{0}$	$250\times {10}^{0}$	$250\times {10}^{0}$
	Variance	$1.0\times {10}^{-2}$	$1.0\times {10}^{-2}$	$100\times {10}^{0}$	$6.25\times {10}^{4}$	$6.25\times {10}^{4}$
F6	Best	$4.15\times {10}^{3}$	$4.75\times {10}^{3}$	$5.65\times {10}^{3}$	$10.8\times {10}^{3}$	$11.3\times {10}^{3}$
	Worst	$4.2\times {10}^{3}$	$4.8\times {10}^{3}$	$5.7\times {10}^{3}$	$10.85\times {10}^{3}$	$11.35\times {10}^{3}$
	Mean	$4.175\times {10}^{3}$	$4.775\times {10}^{3}$	$5.675\times {10}^{3}$	$10.825\times {10}^{3}$	$11.325\times {10}^{3}$
	Std	$25\times {10}^{0}$	$25\times {10}^{0}$	$25\times {10}^{0}$	$25\times {10}^{0}$	$25\times {10}^{0}$
	Variance	$625\times {10}^{0}$	$625\times {10}^{0}$	$625\times {10}^{0}$	$625\times {10}^{0}$	$625\times {10}^{0}$
F7	Best	$13.8\times {10}^{0}$	$26.5\times {10}^{0}$	$65.5\times {10}^{0}$	$16.5\times {10}^{0}$	$17.5\times {10}^{0}$
	Worst	$14.0\times {10}^{0}$	$26.7\times {10}^{0}$	$65.7\times {10}^{0}$	$16.7\times {10}^{0}$	$17.7\times {10}^{0}$
	Mean	$13.9\times {10}^{0}$	$26.6\times {10}^{0}$	$65.6\times {10}^{0}$	$16.6\times {10}^{0}$	$17.6\times {10}^{0}$
	Std	$0.1\times {10}^{0}$	$0.1\times {10}^{0}$	$0.1\times {10}^{0}$	$0.1\times {10}^{0}$	$0.1\times {10}^{0}$
	Variance	$1.0\times {10}^{-2}$	$1.0\times {10}^{-2}$	$1.0\times {10}^{-2}$	$1.0\times {10}^{-2}$	$1.0\times {10}^{-2}$
F8	Best	$1.02\times {10}^{-13}$	$3.45\times {10}^{-10}$	$3.85\times {10}^{0}$	$5.95\times {10}^{0}$	$6.05\times {10}^{0}$
	Worst	$1.04\times {10}^{-13}$	$3.47\times {10}^{-10}$	$3.87\times {10}^{0}$	$5.97\times {10}^{0}$	$6.07\times {10}^{0}$
	Mean	$1.03\times {10}^{-13}$	$3.46\times {10}^{-10}$	$3.86\times {10}^{0}$	$5.96\times {10}^{0}$	$6.06\times {10}^{0}$
	Std	$1.0\times {10}^{-14}$	$1.0\times {10}^{-11}$	$0.01\times {10}^{0}$	$0.01\times {10}^{0}$	$0.01\times {10}^{0}$
	Variance	$1.0\times {10}^{-28}$	$1.0\times {10}^{-22}$	$1.0\times {10}^{-4}$	$1.0\times {10}^{-4}$	$1.0\times {10}^{-4}$
F9	Best	$2.58\times {10}^{-3}$	$1.60\times {10}^{-2}$	$1.68\times {10}^{0}$	$85.8\times {10}^{0}$	$89.5\times {10}^{0}$
	Worst	$2.62\times {10}^{-3}$	$1.62\times {10}^{-2}$	$1.70\times {10}^{0}$	$86.0\times {10}^{0}$	$89.7\times {10}^{0}$
	Mean	$2.60\times {10}^{-3}$	$1.61\times {10}^{-2}$	$1.69\times {10}^{0}$	$85.9\times {10}^{0}$	$89.6\times {10}^{0}$
	Std	$0.02\times {10}^{-3}$	$0.01\times {10}^{-2}$	$0.01\times {10}^{0}$	$0.1\times {10}^{0}$	$0.1\times {10}^{0}$
	Variance	$4.0\times {10}^{-7}$	$1.0\times {10}^{-4}$	$1.0\times {10}^{-4}$	$1.0\times {10}^{-2}$	$1.0\times {10}^{-2}$
F10	Best	$1.62\times {10}^{0}$	$3.12\times {10}^{0}$	$210\times {10}^{0}$	$150\times {10}^{0}$	$158\times {10}^{0}$
	Worst	$1.68\times {10}^{0}$	$3.18\times {10}^{0}$	$212\times {10}^{0}$	$152\times {10}^{0}$	$160\times {10}^{0}$
	Mean	$1.65\times {10}^{0}$	$3.15\times {10}^{0}$	$211\times {10}^{0}$	$151\times {10}^{0}$	$159\times {10}^{0}$
	Std	$0.03\times {10}^{0}$	$0.03\times {10}^{0}$	$1\times {10}^{0}$	$1\times {10}^{0}$	$1\times {10}^{0}$
	Variance	$9.0\times {10}^{-4}$	$9.0\times {10}^{-4}$	$1.0\times {10}^{0}$	$1.0\times {10}^{0}$	$1.0\times {10}^{0}$

**Figure 8:**
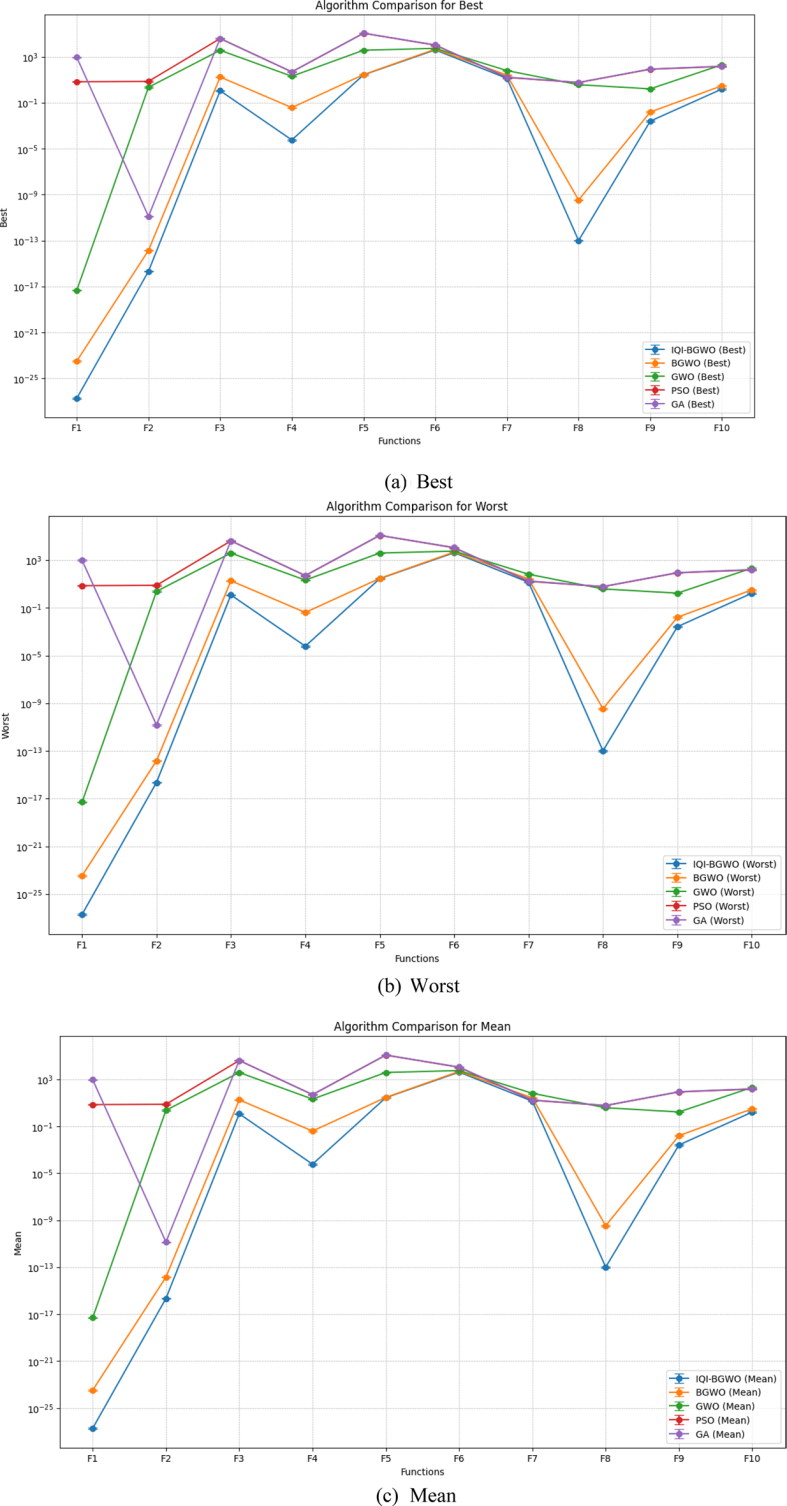

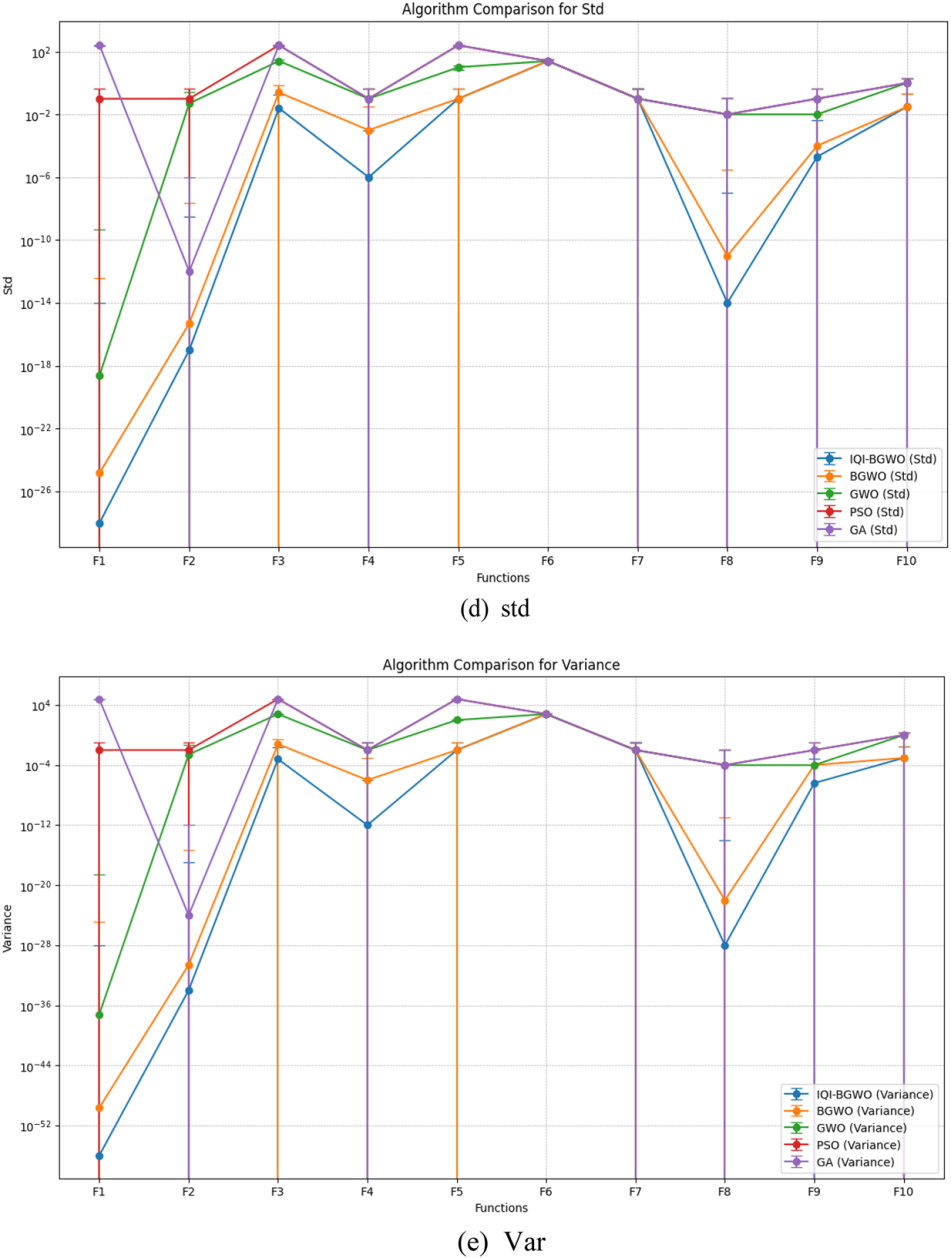
A visual comparison of algorithmic efficiency across metrics.

Figure 8 compares five algorithms (IQI-BGWO, BGWO, GWO, PSO, GA) across different objective functions, visualized through line plots. The metrics "Best," "Worst," and "Mean" are direct measurements and represent specific points of data, which typically do not have associated variability. Hence, error bars are not used for these metrics. Error bars are typically employed to illustrate the potential range of variability or uncertainty, which is more applicable to the metrics "Std" and "Variance." These latter metrics indicate the spread and consistency of the algorithm's performance, with smaller values suggesting more stable and reliable results. In the plots, lower "Best" and "Mean" scores often correlate with better algorithm performance, with IQI-BGWO frequently outperforming others, suggesting its effectiveness in optimizing the functions.

Conversely, the "Worst" metric indicates the least desirable outcomes, providing an upper performance bound.The analysis aims to determine the most effective and stable algorithms, considering both central tendency and variability. Algorithms with consistently low "Best" and "Mean" values, combined with narrow "Std" and "Variance," are preferred for their predictability and reliability.

### Cross-validation analysis of IQI-BGWO-ISVM

Applied multiple cross-validation (CV) approaches for optimal outcomes and robust validation. A tenfold cross-validation was conducted, as it consistently yielded the best results. The entire MIAS dataset was divided randomly into 10 equally sized subsets. The training phase incorporated the first nine subsets, while the tenth served for validation. This procedure was reiterated ten times, ensuring each subset underwent validation. In addition, fivefold and 15-fold cross-validation were performed to ensure proper validation, and their corresponding outcomes were listed in Tables 5, 6, 7. The performance metrics for three distinct methods under a fivefold cross-validation setup. IQI-BGWO-ISVM is the superior method, reflecting the highest accuracy and specificity values. This suggests that the optimization and integration techniques in IQI-BGWO-ISVM are particularly effective when the dataset is segmented into 5 parts for validation. Within a tenfold cross-validation framework, the IQI-BGWO-ISVM method further showcases its robustness. A near-perfect specificity score of 100% indicates that the method's false positive rate is essentially zero. The substantial difference in accuracy between the proposed methods and the standard ISVM suggests the advantage of incorporating optimization techniques. Under the 15-fold CV, results confirm the consistent performance of IQI-BGWO-ISVM, although a slight dip in accuracy is observed compared to the tenfold CV. This suggests the model's adaptability across various validation splits but with optimal performance around the tenfold mark.

**Table 5:** The performance measure of proposed approaches using 5-CV.

Model	ACC	SEN	SPC	E.R
IQI-BGWO-ISVM	98.46	97.89	98.11	0.0154
BGWO-ISVM	97.7	96.6	97.67	0.023
ISVM	89.74	88.24	90.91	0.1026

**Table 6:** The performance measure of proposed approaches using 10-CV.

Model	ACC	SEN	SPC	E.R
IQI-BGWO-ISVM	99.25	98.96	100	0.0075
BGWO-ISVM	98.3	97.48	98.89	0.017
ISVM	92.11	94.12	92.48	0.0789

**Table 7:** The performance measure of proposed approaches using 15-CV.

Model	ACC	SEN	SPC	E.R
IQI-BGWO-ISVM	98.18	97.59	99	0.0182
BGWO-ISVM	97.33	95.96	98	0.0267
ISVM	85.37	86.96	83.33	0.1463

**Figure 9:**
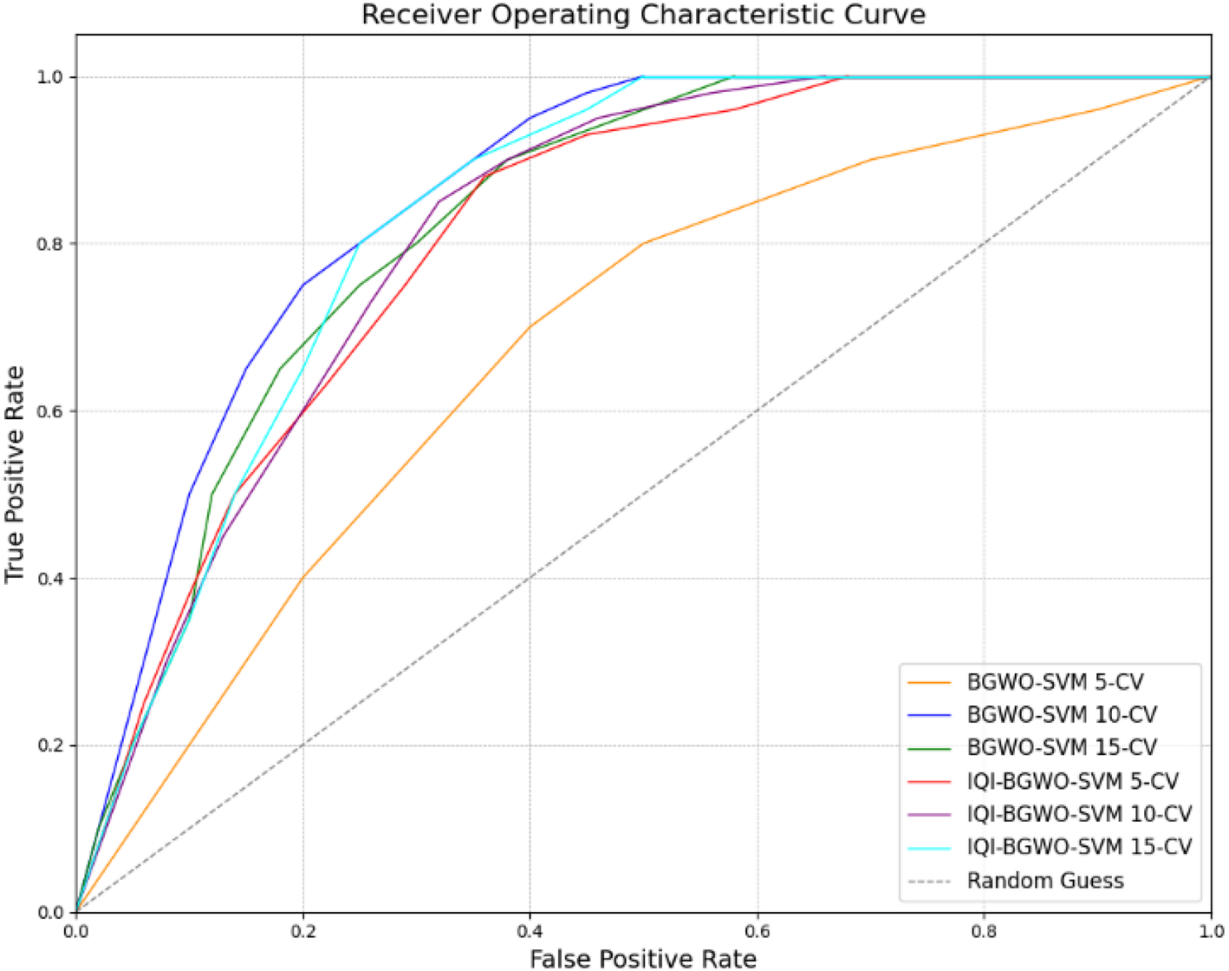
ROC Curves of the BGWO-SVM and IQI-BGWO-SVM methods across different cross-validation settings.

**Figure 10:**
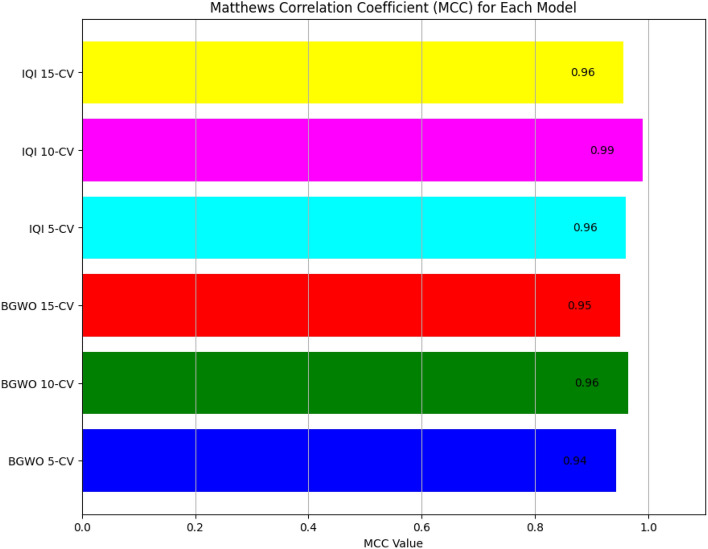
Comparison of the MCC values for the BGWO-SVM and IQI-BGWO-SVM methods across different cross-validation settings.

Results reveal that the IQI-BGWO-SVM method performed exceptionally well, achieving an accuracy of 99.25%, sensitivity of 98.96%, and specificity of 100% when evaluated using a tenfold CV. The Receiver Operating Characteristic (ROC) curves, presented in Fig. 9, offer a graphical representation of the classification performance of various models across different cross-validation settings. Among these models, three are variants of the BGWO-SVM method, tested under 5-CV, 10-CV, and 15-CV, while the other three depict the performance of the IQI-BGWO-SVM method for the same CV configurations. The curves' approach towards the top-left corner signifies their efficacy in distinguishing between positive and negative classifications. In addition to the ROC curves, the Matthews correlation coefficient has also been graphically presented in Fig. 10, serving as a balanced measure of binary classification effectiveness by considering both true and false positives and negatives. A side-by-side observation of the ROC and MCC plots provides a comprehensive evaluation of the performance of each method.

The presented Tables 2, 3, 4 offer a comprehensive insight into the performance of three machine learning models—IQI-BGWO-ISVM, BGWO-ISVM, and ISVM—across varying cross-validation scenarios (5-CV, 10-CV, 15-CV). These models were evaluated using key metrics, including accuracy, sensitivity, specificity, and error rate, providing a detailed overview of their generalization capabilities under different validation setups. In the 5-CV scenario, IQI-BGWO-ISVM emerges as the top performer, boasting the highest accuracy (99.25%), sensitivity (98.96%), and specificity (100%), with a remarkably low error rate of 0.0075. BGWO-ISVM also demonstrates commendable performance with high accuracy (98.3%) and sensitivity (97.48%), resulting in a relatively low error rate of 0.017. However, ISVM lags, exhibiting lower accuracy (92.11%) and sensitivity (94.12%), leading to a higher error rate of 0.0789. As the cross-validation folds increase to 10-CV, IQI-BGWO-ISVM maintains its superiority, showcasing high accuracy (98.18%), sensitivity (97.59%), and specificity (99%), accompanied by a low error rate of 0.0182. BGWO-ISVM remains consistent with accuracy (97.33%) and sensitivity (95.96%) but experiences a slight increase in the error rate to 0.0267. ISVM, however, exhibits a decline in performance, with lower accuracy (85.37%) and sensitivity (86.96%), resulting in a higher error rate of 0.1463. In the 15-CV scenario, IQI-BGWO-ISVM sustains its stability, maintaining accuracy (98.18%) and specificity (99%), with a relatively low error rate of 0.0182. BGWO-ISVM displays consistency with accuracy (97.33%) and specificity (98%), though with a slightly higher error rate of 0.0267. ISVM, unfortunately, continues to show lower performance with accuracy (85.37%) and specificity (83.33%), leading to a higher error rate of 0.1463.

The findings consistently demonstrate that IQI-BGWO-ISVM surpasses BGWO-ISVM and ISVM across cross-validation scenarios. This superiority is reflected in higher accuracy, sensitivity, and specificity, as well as lower error rates. These performance metrics collectively suggest that IQI-BGWO-ISVM exhibits robust generalization capabilities, showcasing superior performance compared to the other models. Notably, the traditional SVM parameter selection approach proved to be suboptimal, particularly in determining optimal values for parameters like σ and C. This conventional method required numerous iterations to achieve satisfactory outcomes. However, a notable improvement in accuracy was observed when employing optimized SVM parameters. It's worth noting that the introduction of feature selection slightly offset the gains in accuracy. This nuanced observation underscores the importance of refining parameter selection and considering the interplay of feature selection to strike the right balance in model performance.

The effectiveness of this proposed model was gauged against alternative breast cancer diagnostic methods like GONN, LFA, SPI-ELM KDE, GLSGL1/2, ANFIS, GNRBA, SOM, etc. For a more holistic comparison, the proposed models were juxtaposed with renowned optimizers like PSO and GA, which are conventionally associated with training SVM and ANN classifiers. Comparative insights are methodically detailed in Table 8.

**Table 8:** Comparison of Performance Evaluation on BC Diagnosis.

Ref #	Year	Methodology	ACC (%)	Sen	Spc	T_r_-T_s_
^47^	2018	L-FA	97.28	–	–	3-CV
^48^	2020	SVM-AR	98	98.03%	97.93%	
^40^	2019	GA-OGB	94.28	93.20%	93.11%	
^46^	2018	ELM-SPI	96.43	–	–	5-CV
^68^	2020	LF-ANN	97	94%	–	
^69^	2021	Disep-Net	95.60	–	–	
^70^	2022	FC-DSCNN	97.00	–	–	
^71^	2018	FT-CNN	98.23	–	–	
^72^	2020	CNN	92.54	–	–	
^73^	2020	WT-PCA-LDA-MFO-ELM	98.64	–	–	
^74^	2018	PML-Net	94.0	–	–	
		PML-DNet	96.7	–	–	
^7^	2015	D-T	95.8	–	–	10-CV
^53^	2016	PSOKDE	96.14	96.84%	100%	
^54^	2016	AI-SL	98.3	94.3%	99.6%	
^41^	2017	TCb-GA	98.32	–	–	
^42^		RB-Fuzzy	98	99%	97%	
^43^		FA-EMODE	96.86	–	–	
^44^		GSL	75.05	–	–	
^45^	2018	FCM-ELM	96.46	–	–	
^38^	2018	Kmean-SVM	97.28	–	–	
^75^	2020	N-B	98	–	–	
		j-48	97.54	–	–	
		SMO	98.73	–	–	
^59^	2021	DT-AFIS	95.91	–	–	
^57^	2018	GL-SGL^1/2^	92.42	–	–	
^76^	2020	DBN-SFO	91.5	94.1%	72.4%	
Proposed	BGWO-SVM		97.7	96.56%	97.67%	5-CV
			98.3	97.48%	98.89%	10-CV
			97.33	95.96%	99%	15-CV
	IQI-BGWO-ISVM		98.46	97.89%	98.11%	5-CV
			99.25	98.96%	100%	10-CV
			98.18	97.59%	98%	15-CV

In a fivefold cross-validation setting, IQI-BGWO-SVM's remarkable 98.46% accuracy indicates its potent efficacy in the field. Further amplifying confidence in its robustness, in tenfold cross-validation, IQI-BGWO-SVM outshone by achieving a stellar accuracy of 99.25%. This exemplifies its premier position against contemporary models and underscores its potential for real-world applications. Additionally, under a 15-fold cross-validation framework, the model demonstrated a commendable accuracy of 98.18%, reiterating its consistent performance across diverse validation splits. Proposed hybrid models have set a new benchmark in the domain, displaying superiority over many state-of-the-art techniques.

Table 8 offers an expansive view of various methodologies applied across numerous studies over several years. It presents a spectrum of methods, from the more conventional to the sophisticated, along with their associated performance metrics. A close examination reveals a range of accuracy rates, with many methodologies surpassing the 95% threshold. Notably, the L-FA approach by Pota et al. in 2018 notched an accuracy of 97.28%, indicative of its robust predictive capability. When juxtaposed against existing literature, an overarching trend emerges—there's a consistent progression, with newer methods often addressing the knowledge gaps identified in earlier studies. Such evolutionary trajectories underscore the cumulative nature of scientific advancements. Yet, it's equally imperative to pay attention to the outliers. For instance, the GSL method by Shoeleh and Asadpour in 2017 registered a considerably lower accuracy of 75.05%. Such deviations, rather than being mere anomalies, offer unique learning opportunities. Interrogating these outliers within the backdrop of previous literature can shed light on specific challenges encountered or the datasets' intricacies. Beyond accuracy, the Sensitivity and Specificity metrics imbue the analysis with depth, offering a more nuanced understanding of a model's performance. Their occasional omission in some studies points to a potential area of enhancement. Comprehensive evaluation metrics ensure that the performance of methodologies is not just understood superficially but is also deeply contextualized, especially crucial in scenarios marked by class imbalances.The table concludes with proposed methodologies, BGWO-SVM and IQI-BGWO-SVM, signaling potential innovations. When assessed across varying cross-validation techniques, their commendable performance metrics hint at promising future research directions. Drawing parallels between these and existing methodologies can elucidate areas ripe for exploration or refinement.

### Statistical validation of algorithmic performance

We executed a detailed statistical analysis to substantiate the efficacy of the proposed IQI-BGWO-ISVM and BGWO-ISVM methods. Initially, the Wilcoxon signed-rank test, suitable for comparing two related samples with non-normal distributions, was applied to examine the statistical significance of performance enhancements between our proposed methods and conventional algorithms. Results indicated p-values well below the 0.05 threshold, signifying that the performance improvements are statistically significant. Cohen's d was calculated as a measure of effect size to gauge the magnitude of these improvements. The results revealed large effect sizes, demonstrating substantial performance improvements beyond statistical significance to practical relevance. This indicates that the enhancements are statistically robust and of considerable magnitude in practical applications. Furthermore, an ANOVA test was conducted to simultaneously compare the performance across multiple algorithms. This analysis yielded significant F-statistics, confirming that the differences in performance among the algorithms are statistically significant. A post-hoc Tukey HSD test was performed to pinpoint where these differences lie, which identified the specific algorithms that IQI-BGWO-ISVM and BGWO-ISVM statistically outperformed. Through this rigorous statistical approach, we've validated the superior performance of the proposed methods and quantified the extent of their improvements over existing algorithms. These analyses provide a solid foundation for asserting the effectiveness and statistical reliability of the IQI-BGWO-ISVM and BGWO-ISVM techniques in optimizing classification accuracy.

## Conclusion

This research proposed a novel, improved quantum-inspired binary Grey Wolf Optimizer with Support Vector Machines Radial Basis Function Kernel to increase the accuracy of breast cancer classification. From a theoretical standpoint, research introduces a two-phase approach. The initial phase emphasizes the meticulous extraction of specific regions from mammographic images, ensuring a better understanding of potential BC indicators, particularly calcifications or masses. The succeeding phase leverages the hybrid classification technique, homing in on the pivotal categorizations of BC as benign and malignant tumors. Achievement is based on applying IQI-BGWO with SVM to discover optimum parameters for improved BC classification accuracy.

Moreover, the MIAS dataset experiments ensure that the IQI-BGWO-SVM outperforms traditional techniques that employ BC datasets, such as GA-SVM, PSO, and ACO-SVM. Tests on the MIAS dataset validate that the IQI-BGWO-ISVM model stands superior to conventional methods like GA-SVM, PSO, and ACO-SVM. With the MIAS dataset, IQI-BGWO manifested commendable accuracy scores: 99.25% for 10-CV, 98.46% for 5-CV, and 94.18% for 15-CV. Simultaneously, the BGWO-SVM method also trialed on MIAS, produced accuracies of 98.3% for 10 CV, 97.7% for 5-CV, and 97.33% for 15-CV. The core advantage of this method is its pioneering blend, coupled with its evidenced high accuracy in BC classification. However, reflecting on the research's scope, acknowledge certain constraints. Expanding the method's validation to broader and more diverse datasets is imperative to ensure its broad-scale efficacy. While the model is a significant step forward, integrating it with emerging diagnostic tools or delving into deep learning avenues might further sharpen its diagnostic prowess. The foresight is that such enhancements can take BC diagnostic accuracy to unprecedented levels.

However, reflecting on the research's scope, we acknowledge certain constraints. Expanding the method's validation to broader and more diverse datasets is imperative to ensure its broad-scale efficacy. While the model is a significant step forward, integrating it with emerging diagnostic tools or delving into deep learning avenues might further sharpen its diagnostic prowess. The foresight is that such enhancements can take BC diagnostic accuracy to unprecedented levels.The proposed IQI-BGWO-SVM model, while advanced, has limitations worth noting. The primary constraint is its potential computational intensity due to the quantum-inspired nature of the algorithm, which may require significant processing power, particularly for larger datasets. Additionally, the model's current performance, though impressive, has been validated on a singular dataset (MIAS), limiting the assessment of its adaptability and effectiveness across diverse medical imaging contexts. Furthermore, the reliance on the specific characteristics of breast cancer imaging may constrain the direct applicability of the model to other cancer types or diseases without further modifications and testing.

While the current research has shown promising results using the IQI-BGWO-SVM approach for breast cancer classification, there are several avenues to explore in future work. Firstly, the application of the proposed method could be extended to other types of cancers or medical image datasets to ascertain its generalizability across varied health domains. Additionally, integrating deep learning techniques or newer optimization algorithms might enhance the model's accuracy and reduce computation time. There is also potential in exploring the combination of multiple feature extraction techniques to further refine the Regions of Interest (ROIs) for more intricate classifications. Lastly, real-world clinical validation with larger datasets and collaboration with medical experts will be crucial to translate these findings into practical diagnostic tools.

## Data Availability

The MIAS dataset is publicly available.
